# Colanic acid-mediated phage resistance enhances virulence in high-risk global clone *Escherichia coli* ST410

**DOI:** 10.1371/journal.ppat.1013807

**Published:** 2025-12-22

**Authors:** Jieying Tu, Jun Yang, Kewei Song, Jinghan Wang, Nuoyan Zhang, Wanyun He, Jinghao Liu, Zhongpeng Cai, Yuman Bai, Luchao Lv, Bingqing Zhu, Pan Tao, Jie Feng, Jian-Hua Liu

**Affiliations:** 1 State Key Laboratory for Animal Disease Control and Prevention, Key Laboratory of Zoonosis of Ministry of Agricultural and Rural Affairs, College of Veterinary Medicine, South China Agricultural University, Guangzhou, China; 2 Guangdong Provincial Key Laboratory of Pharmaceutical Bioactive Substances, School of Basic Medical Sciences, Guangdong Pharmaceutical University, Guangzhou, China; 3 State Key Laboratory of Agricultural Microbiology, College of Veterinary Medicine, Huazhong Agricultural University, Wuhan, Hubei, China; 4 State Key Laboratory of Microbial Resources, Institute of Microbiology, Chinese Academy of Sciences, Beijing, China; University of Utah, UNITED STATES OF AMERICA

## Abstract

The global rise of multidrug-resistant *Escherichia coli* ST410 presents a growing clinical threat. We isolated a highly lytic phage targeting ST410, but rapid resistance emerged both *in vitro* and *in vivo*. Beyond receptor mutations, we identified a hypermucoid mutant that evades phage infection primarily through colanic acid overproduction, providing broad phage resistance. This colanic acid overproduction also led to diminished macrophage phagocytosis *in vitro* and accelerated mortality in mice. Transcriptomic and genetic analyses linked this phenotype to activation of the Rcs pathway. Notably, naturally occurring ST410 isolates with colanic acid-overproducing phenotypes were also observed, suggesting that capsule-mediated immune evasion may arise in clinical populations. To overcome resistance, we designed a rational four-phage cocktail with diverse receptors, achieving robust therapeutic efficacy in infection models. Our findings highlight that phage resistance can be accompanied by enhanced virulence potential through capsule-mediated immune evasion, with colanic acid playing a central role, emphasizing important considerations for phage therapy design.

## Author summary

Multidrug-resistant *Escherichia coli* ST410 has emerged as a high-risk global clone threatening both human and animal health. Phages offer promising alternatives to antibiotics, yet the rapid evolution of resistance limits their therapeutic utility. Here, we isolated a lytic phage targeting ST410 and discovered that bacteria evade infection by hyperproducing colanic acid, a capsule exopolysaccharide. This mutation conferred broad phage resistance and, importantly, enhanced immune evasion and accelerated disease progression. This phenotype appears to increase virulence potential. We also identified naturally occurring hypermucoid ST410 clinical isolates displaying similar capsule-associated traits, suggesting this evolutionary trajectory may already exist in real-world settings. To counter resistance, we designed a four-phage cocktail with broad host coverage and robust therapeutic efficacy. Our findings illustrate that phage resistance can carry virulence-associated consequences, emphasizing the need to incorporate evolutionary principles into phage therapy strategies. The emergence of hypermucoid phenotypes in pathogenic *E. coli* lineages may pose an underappreciated risk in clinical and veterinary settings, underscoring the importance of monitoring capsule-related evolution and carefully evaluating safety during phage therapy.

## Introduction

Antimicrobial resistance (AMR) in bacterial pathogens is a major public health threat in the 21st century. A recent study in *The Lancet* estimates that antimicrobial-resistant infections could cause over 39 million deaths globally within the next 25 years [1]. With limited effective antibiotics available, innovative strategies are urgently needed to protect human health and address this therapeutic crisis, with phage therapy re-emerging as a promising strategy [2–4].

However, a major challenge in phage therapy is the frequent emergence of phage-resistant bacterial variants during treatment. Phage-host dynamics are largely governed by interactions with bacterial surface structures—including capsules, outer membrane proteins (OMPs), and lipopolysaccharides (LPSs)—which serve as primary adsorption sites [5]. Genetic or structural modifications of these receptors can confer resistance by preventing phage binding, but such adaptations often incur evolutionary trade-offs, such as reduced virulence or altered antibiotic susceptibility [6]. Among these structures, capsular polysaccharides frequently act as a first line of defense, playing dual roles in immune evasion and resistance to phage predation [7,8]. In encapsulated bacteria, phage-driven selection often results in capsule loss or reduction, leading to predictable fitness costs due to the capsule’s pleiotropic roles in host-pathogen interactions—a phenomenon commonly referred to as an evolutionary trade-off [9–13]. However, non-encapsulated bacteria, owing to their diverse surface architectures, can evolve phage resistance through multiple distinct strategies. Yet, the fitness trade-offs associated with these adaptations remain poorly understood. Elucidating how such trade-offs arise during phage therapy is critical, as they can profoundly influence treatment efficacy and the long-term success of phage-based interventions.

*Escherichia coli,* with inherently low capsule expression, offers a valuable model for exploring phage-driven evolution in non-encapsulated pathogens. Within *Escherichia coli*, certain lineages have emerged as so-called high-risk clones, attracting increasing attention due to their alarming clinical and epidemiological features. These strains are characterized by a close common phylogenetic origin, global dissemination, enhanced ability to colonize, spread, and persist in a variety of niches, increased pathogenicity or antibiotic resistance, and remarkable adaptability, often resulting in severe or recurrent infections [14]. Among its many lineages, *E. coli* ST410 is a globally disseminated, multidrug-resistant high-risk clone capable of colonizing diverse ecological niches, including humans, animals, and the environment [14–17]. In recent years, a novel subclone of ST410, designated B5/H24RxC, has emerged, marked by a shift in O-antigen composition and the acquisition of a high pathogenicity island (HPI). These genetic changes distinguish it from earlier B4/H24RxC lineages, indicating ongoing evolution toward enhanced virulence [17]. Furthermore, the recent rise of ST410 as the predominant zoonotic clone among carbapenem-resistant *E. coli* in companion animals highlights the lineage’s ecological adaptability and public health relevance [18]. The close human-animal contact facilitates bidirectional transmission of such high-risk strains, underscoring the need for coordinated control strategies [19–21]. Despite phages’ narrow host ranges, they hold therapeutic potential against clonally related lineages, as shown in successful examples like the Katrice-16 cocktail for *Klebsiella pneumoniae* ST16 and region-specific formulations for carbapenem-resistant *Acinetobacter baumannii* [2,4]. Accordingly, phages represent a promising yet undeveloped strategy for treating *E. coli* ST410 infections, with strong potential for personalized therapy in patients and companion animals.

In this study, we isolated a lytic phage targeting *E. coli* ST410, but resistance emerged rapidly under selective pressure. We identified a hypermucoid mutant in which colanic acid overproduction primarily conferred broad phage resistance and was associated with an increased virulence potential, likely driven by enhanced immune evasion. This work provides mechanistic insights into how capsule-mediated immune evasion can shape phage-bacteria dynamics and informs the rational design of phage therapy strategies, taking into account potential virulence-associated consequences during early host-phage interactions.

## Results

### Phage isolation, characterization, and therapeutic evaluation

**Fig 1:**
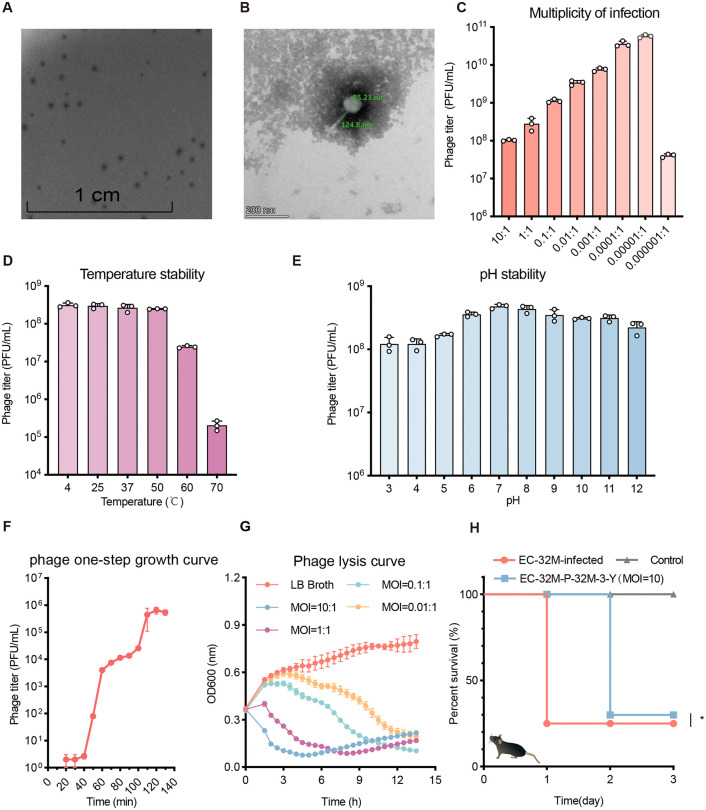
Morphology, biological properties, and therapeutic potential of phage P-32M-3-Y. **A.** Plaque morphology of phage P-32M-3-Y. **B.** Transmission electron microscopy (TEM) image of P-32M-3-Y, revealing its typical morphology. **C.** Phage yield under different multiplicities of infection (MOIs). **D.** Thermal stability profile of P-32M-3-Y. **E.** pH stability of P-32M-3-Y across a range of conditions. **F.** One-step growth curve illustrating the latent period and burst size of the phage. **G.** *In vitro* bactericidal activity of P-32M-3-Y against *E. coli* ST410. The initial bacterial suspension had an OD₆₀₀ of ~0.4, corresponding to approximately 10⁸ CFU/mL. **H.** *In vivo* therapeutic efficacy of phage P-32M-3-Y in a murine infection model. Mice were intraperitoneally challenged with 10⁸ CFU of the EC-32M strain, corresponding to the previously determined median lethal dose (LD₅₀). Group sizes: EC-32M-infected (n = 8), EC-32M-P-32M-3-Y (n = 10), Control (n = 6). Survival was monitored for 3 days post-infection. Statistical analysis was performed using the Gehan-Breslow-Wilcoxon test.

The carbapenem-resistant *E. coli* ST410 strain EC-32M, isolated from a canine patient, was used to isolate phage P-32M-3-Y from wastewater, showing as a member of the *Myoviridae* family by transmission electron microscopy (TEM) (Fig 1A and 1B). Whole-genome sequencing classified P-32M-3-Y as an *Agtrevirus* within the *Ackermannviridae* family, with no detected integrase, toxin, or virulence genes (S5 Fig), supporting its therapeutic potential [22]. P-32M-3-Y exhibited high environmental stability (pH 3–12; 4–50 °C), an exceptionally low optimal MOI (10 ⁻ ⁵), and a lytic cycle characterized by a 40-minute latent period, a 70-minute burst period, and a burst size of 108 ± 10 PFU/cell (Fig 1C–1F). Host range profiling revealed that P-32M-3-Y specifically lysed ST410 strains carrying the novel O-antigen variant Onovel1(24/25) (Table 1), implicating this structure as its receptor. Notably, although strain GDG24AEC-39-1PT harbors the Onovel11 O-antigen like other susceptible isolates, it was not lysed by P-32M-3-Y. The reason for this resistance will be explained later in the Results section.

**Table 1:** The host range of phage P-32M-3-Y against *Escherichia coli* ST410. + , P-32M-3-Y could form a clear zone or plaque; -, P-32M-3-Y could not form a clear zone or plaque.

Subgroup	Strains	O-antigen	Lysis ability
B3/H24Rx	AHM7C101I, AHM21C104I, AHM7C52I, AHM7C60I, AHM7C62I, AHM7C66I, AHM7C75I, AHM7C79I, AHM7C80I, AHM7C82I, AHM7C85I, AHM7C86I, AHM7C87I, AHM7C89I, AHM7C95I, AHM7C99I, XJCJ22B18, XJCJ22B4,	Onovel1	+
	AHM8C12AI	O8	–
	GYX208DH5–2	O61	–
	AHM9C239I	O76	–
	XJCJ20B32, XJCJ20N57	Onovel2	–
B4/H24RxC	GZA9A29M, JXZ9A1M, SHC9A30M	O8	–
B5/H24RxC	GYX208DH6–1, JXZ9A11M, JXZ9A43M, JXZ9A45M, JXZ9A6M, EC-CRE564	Onovel1	+
	GDG24A-39-1PT	Onovel1	–

*In vitro* time-kill assays demonstrated rapid bacterial clearance within 3 hours, but regrowth due to phage-resistant mutants emerged by 5 hours (Fig 1G). In a murine peritoneal infection model, phage treatment modestly delayed mortality yet failed to prevent death in 70% of mice (Fig 1H). These findings underscore that, despite favorable in biological characteristics, the therapeutic efficacy of P-32M-3-Y is compromised by the rapid emergence of phage resistance.

### Phage resistance analysis and receptor identification

**Fig 2:**
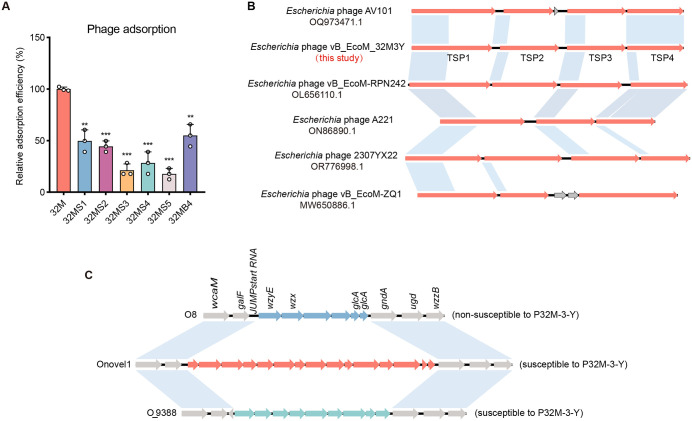
Phage resistance analysis of O-antigen mutants. **A.** Relative adsorption efficiency of phages on O-antigen mutant strains. Values were determined by unpaired two-tailed *t*-test. “ns” indicates no significant difference (*P* > 0.05); “*”, “**”, and “***” denote significant (*P* < 0.05), highly significant (*P* < 0.01), and extremely significant (*P* < 0.001) differences, respectively. **B.** Sequence comparison of tail spike proteins from phages belonging to the genus *Agtrevirus*. Arrows represent open reading frames (ORFs), and blue blocks indicate homologous nucleotide regions (≥99.0% identity) in the same orientation. Branch lengths are drawn to scale. **C.** Genomic comparison of O-antigen gene clusters O8, Onovel1, and O_9388. Arrows represent open reading frames (ORFs). ST410 harbors more than 70 O-antigen types, with O8 accounting for approximately 48% of ST410 O-antigens in the NCBI database. O8 is the most prevalent O-antigen in ST410, but it is insensitive to phage P-32M-3-Y. Onovel1, a novel O-antigen in ST410, and O_9388, an untyped O-antigen in ST9388, are both sensitive to phage P-32M-3-Y.

To elucidate the limited therapeutic efficacy of P-32M-3-Y, phage-resistant clones were isolated from *in vitro* high-MOI co-cultures and infected murine tissues (S1 Fig) and subjected to whole-genome sequencing. Comparative analyses revealed that 13 of 14 resistant isolates carried disruptive mutations within the O-antigen biosynthesis locus (*GMDAJ_09155*, *GMDEAJ*_*00370*, *GMDEAJ*_*09190*, *GMDEAJ*_*09145*), predominantly resulting in premature termination codons (Table 2). One isolate, EC-32M3BB, was obtained from the *in vitro* co-cultures of *E. coli* EC-32M and phage P-32M-3-Y and harbored a T → A transversion in *yrfF*, a known negative regulator of the RcsCDB phosphorelay. Given the enrichment of mutations in O-antigen-associated genes, we hypothesized that the O-antigen serves as the principal receptor for P-32M-3-Y [23]. Supporting this, resistant isolates exhibited markedly reduced phage adsorption efficiency (44.94%–82.24%) compared to the parental EC-32M strain (Fig 2A).

**Table 2:** Summary of identified phage resistance mutations.

Resistant isolate	Gene or Locus tag	Putative gene function	Nucleotide Mutation	Amino Acid Mutation
32MS4	*GMDEAJ_09155*	Glycosyltransferase family 8 protein	Deletion (541delG)	Gly181fsTer186
32MB4	*GMDEAJ_09155*	Glycosyltransferase family 8 protein	Deletion (700delA)	Glu234fsTer264
32MS1,32M1P4	*GMDEAJ_09155*	Glycosyltransferase family 8 protein	Deletion (746delA)	Lys249fsTer264
32MS3,32MS5	*GMDEAJ_09155*	Glycosyltransferase family 8 protein	Deletion (828delT)	Ser276fsTer285
32M1G22	*GMDEAJ_00370*	Glycosyltransferase	Deletion (693_704delATATAATAAATG)	Tyr232_Lys234del
32M1P18	*GMDEAJ_09190*	Glycosyltransferase	Insertion (6_12insAATTTCT)	Gln2fsTer21
32M10G13,32M10S41	*GMDEAJ_09145*	O-antigen polymerase	Deletion (827_828delAT)	Asn276Ter282
32M12P8	*GMDEAJ_09145*	O-antigen polymerase	Deletion (912delT)	Ala304Ter311
32MS2	*GMDEAJ_09145*	O-antigen polymerase	Deletion (1058delT)	Val353Ter376
32M10G34	*GMDEAJ_09145*	O-antigen polymerase	Point mutation (1062T > A)	Phe355Leu
EC-32M3BB	*yrfF*	YrfF	Point mutation (292T > A)	Tyr97Asn

Annotation of the P-32M-3-Y genome identified four distinct tail spike proteins (TSP1–TSP4), each possessing a conserved N-terminal domain and hypervariable C-terminal regions (Fig 2B), characteristic of *Agtrevirus* phages known for multi-receptor specificity [24]. Host range assays further showed that P-32M-3-Y lysed *E. coli* ST9388 strains expressing an uncharacterized O-antigen (O_9388) in addition to ST410 strains bearing the Onovel1 serotype (Fig 2C and S1 Table). Together, these data suggested that P-32M-3-Y exploits multiple O-antigen variants via distinct TSPs, which contributed to its expanded host tropism compared to single-receptor phages [25].

### Enhanced colanic acid production confers broad phage resistance via YrfF mutation

**Fig 3:**
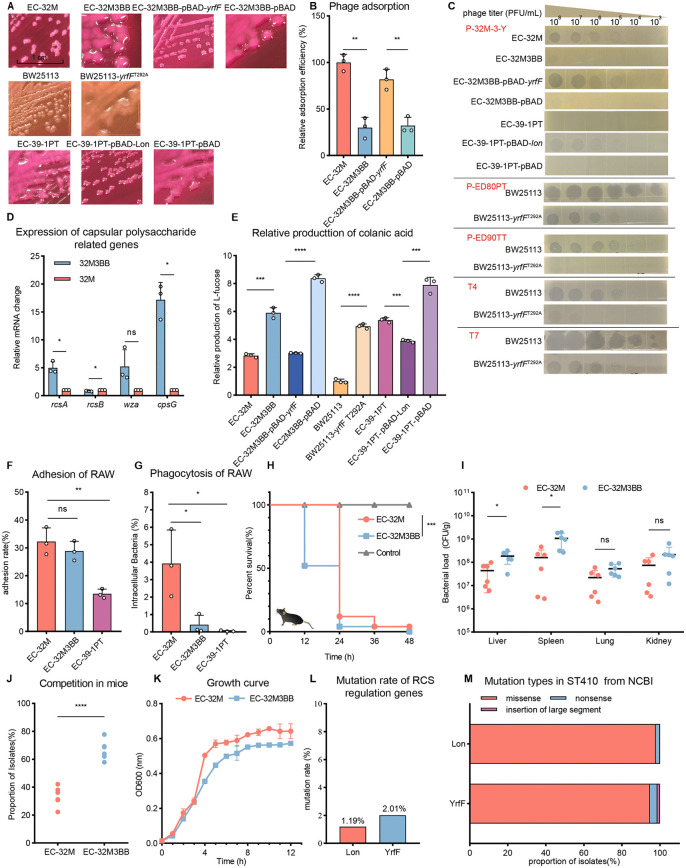
Colanic acid–mediated phage resistance via *yrfF* mutation drives immune evasion and hypervirulence in *E. coli* ST410. **A.** Colony morphology of wild-type and mutant strains: EC-32M, EC-32M3BB, EC-32M3BB-pBAD, EC-32M3BB-pBAD-yrfF, BW25113, BW25113-*yrfF*^T292A^, EC-39-1PT, EC-39-1PT-pBAD, and EC-39-1PT-pBAD-Lon. **B.** Relative phage adsorption efficiency of EC-32M, EC-32M3BB, EC-32M3BB-pBAD, and EC-32M3BB-pBAD-*yrfF*. Values were determined by unpaired two-tailed t-test. “ns” indicates no significant difference (P > 0.05); “*”, “**”, and “***” denote significant (*P* < 0.05), highly significant (*P *< 0.01), and extremely significant (*P* < 0.001) differences, respectively. **C.** Phage sensitivity assays showing resistance profiles of *yrfF*^T292A^ mutant and complemented strains against various phages. **D.** Differential expression of capsule-related genes in EC-32M and EC-32M3BB, as determined by qRT-PCR. Values were determined by unpaired two-tailed t-test. **E.** Quantification of colanic acid production in different *E. coli* strains. The amount of colanic acid was normalized relative to that of *E. coli* BW25113, which exhibited the lowest production and was therefore set as the baseline (1.0). Data represent means ± SD from three biological replicates. Values were determined by unpaired two-tailed t-test. **F.** Adhesion of EC-32M, EC-32M3BB, and EC-39-1PT to RAW264.7 macrophages. RAW264.7 cells were infected with EC-32M, EC-32M3BB or EC-39-1PT (MOI = 10) for 1.5 h. Adhesion was quantified by plating cell-associated bacteria, expressed as percentage of the initial inoculum. Data are mean ± SD (n = 3 independent experiments). *P* values were determined by unpaired two-tailed *t*-test. **G.** Phagocytosis of EC-32M, EC-32M3BB, and EC-39-1PT by RAW264.7 murine macrophages. RAW264.7 cells were infec*t*ed with EC-32M or capsule-overproducing EC-32M3BB (MOI = 10) for 2 h, followed by ampromycin treatment to eliminate extracellular bacteria. Internalized bacteria were quantified by plating cell lysates, expressed as the percentage of cell-associated bacteria. Data are mean ± SD (n = 3 independent experiments). *P* values were determined by unpaired two-tailed *t*-test. H Survival curves of mice infected wi*t*h EC-32M or EC-32M3BB in an intraperitoneal infection model. The dosage of bacterial is 5 × 10^7^CFU. Group size: n = 25 for EC-32M and EC-32M3BB, n = 6 for Control. Statistical analysis was performed using the Gehan-Breslow-Wilcoxon test. **I.** Bacterial burden in major organs (liver, spleen, kidney, lung) of infected mice. The dosage of bacterial is 5 × 10^7^CFU. Group size: n = 6 for each group. *P* values were determined by unpaired two-tailed *t*-test. **J.** *In vivo* competi*t*ion between EC-32M and EC-32M3BB in mice. C57BL/6J mice (n = 6) were intraperitoneally co-infected with a 1:1 mixture of EC-32M and EC-32M3BB (total 5 × 10^7^ CFU). The input ratio values across biological replicates were 1.08, 0.96, and 1, indicating <0.1 log variation from the intended 1:1 ratio. Blood samples were collected from the tail vein for CFU enumeration on selective media at 24 h post-infection. Relative abundance was calculated as the proportion of total bacteria. Data are mean ± SD; *P* values by paired two-tailed *t*-test. **K.** Growth kinetics of EC-32M and EC-32M3BB *in vitro*. **L.** Prevalence of mutations in Rcs regulatory proteins (Lon, YrfF) among 3,784 *E. coli* ST410 genomes from NCBI. M. Distribution of missense, nonsense, and insertion mutations in *yrfF* and *lon* among *E. coli* ST410 genomes from NCBI. Mutations in *yrfF* were detected in 76 isolates (2.01%), including one large nucleotide insertion, 72 missense, and 3 nonsense mutations. Mutations in *lon* were found in 45 isolates (1.19%), comprising 44 missense and 1 nonsense mutation. Percentages are calculated relative to the total number of isolates analyzed.

To elucidate the mechanism underlying phage resistance in *E. coli* ST410, we focused on the mutant strain EC-32M3BB, which displayed a distinct and stable mucoid colony morphology not observed in other resistant isolates (Fig 3A). Whole-genome sequencing revealed that EC-32M3BB lacked mutations in the O-antigen biosynthesis cluster—commonly associated with receptor-mediated resistance—and instead harbored a unique T292A point mutation in *yrfF*. Similar to the O-antigen mutants, we first assessed phage adsorption efficiency and found that EC-32M3BB exhibited ~70% reduced binding to phage P-32M-3-Y compared to the parental strain EC-32M (Fig 3C). Complementation of *yrfF* via plasmid pBAD24 reversed the mucoid phenotype (Fig 3A), restored phage adsorption to near-wild-type levels (Fig 3B), and resensitized the strain to phage infection (Fig 3C). This result confirms that the *yrfF* T292A mutation confers phage resistance by promoting mucoid overproduction, which in turn impairs phage adsorption.

Next, we performed transcriptomic analysis to investigate the differences between the two strains (S2 Fig). KEGG enrichment identified upregulation of exopolysaccharide biosynthesis pathways (S2B Fig). Given that *yrfF* is a negative regulator of the Rcs phosphorelay controlling colanic acid biosynthesis [26], we examined transcriptional changes within the capsule operon. Key genes in the colanic acid cluster—including *rcsA*, *cpsG*, *wcaF*, *wcaA*, *wcaL*, and *wcaE*—were upregulated by 2.15- to 8.06-fold (S2C Fig). qRT-PCR revealed strong upregulation of *cpsG* (17.16 ± 3.11-fold), *wza* (5.22 ± 2.99-fold), and *rcsA* (4.98 ± 1.09-fold), the latter being a key activator of colanic acid biosynthesis (Fig 3D). In contrast, the expression of *rcsB*, the response regulator of the RcsCDB phosphorelay, was slightly decreased in EC-32M3BB (0.85 ± 0.06-fold), yet this reduction was statistically significant due to low intra-group variability. These results suggest that the *yrfF* T292A mutation enhances capsule production not through increased transcription of *rcsB*, but might likely via altered RcsB phosphorylation status which need further investigation. Together, these findings identify residue T292 of YrfF as a key modulator linking the Rcs pathway to capsule overproduction and phage resistance.

We next quantified colanic acid production by measuring L-fucose, one of its major components. The EC-32M3BB mutant produced over twice as much L-fucose as the parental strain EC-32M, while complementation with wild-type *yrfF* restored L-fucose levels to wild type (Fig 3E). These results confirm that the YrfF T292A mutation in EC-32M3BB drives colanic acid overproduction, which in turn mediates phage resistance.

To test the causal role of this mutation, we introduced the *yrfF* T292A substitution into the model strain *E. coli* BW25113 —a well-characterized laboratory strain with defined genetic background and known phage susceptibility. This model system allowed us to assess the phenotypic consequences of the mutation in a clean and reproducible context. The resulting mutant exhibited a mucoid colony morphology (Fig 3A), with L-fucose levels 4.94-fold higher than those of the parental BW25113 strain (Fig 3E) similar to that of the naturally evolved EC-32M3BB strain, indicating successful activation of colanic acid biosynthesis. We then tested the susceptibility of the mutant to four lytic phages: PED80PT and PED90TT (both isolated from duck farm wastewater), and the model phages T4 and T7. All four phages efficiently infected wild-type BW25113, but their lytic activity was substantially reduced or abolished in the *yrfF* T292A mutant (Fig 3C), as indicated by diminished or absent plaque formation. These results confirm that the T292A mutation confers broad, strain-independent phage resistance likely mediated through receptor masking by colanic acid overproduction.

### Capsule hyperproduction increases both phage resistance and virulence

Given the central role of bacterial capsules in immune evasion, we examined whether colanic acid overproduction enhances virulence in parallel with phage resistance. Adhesion and phagocytosis assays were performed using RAW264.7 macrophages at a multiplicity of infection (MOI) of 10. The capsule-overproducing mutant EC-32M3BB showed a comparable adhesion rate to the parental EC-32M strain (28.87% vs. 32.25%) (Fig 3F), indicating only a modest reduction in macrophage binding. In contrast, phagocytosis efficiency was markedly reduced in EC-32M3BB, with only 0.42% of bacteria internalized compared to 4.92% for EC-32M (Fig 3G). These data suggest that the enhanced resistance to phagocytic clearance in EC-32M3BB is primarily attributable to immune evasion rather than reduced attachment.

In a murine abdominal infection model, mice infected intraperitoneally with 5 × 10^7^ CFU of EC-32M3BB displayed earlier onset of disease and more rapid progression to mortality. Specifically, ~ 50% of mice infected with EC-32M3BB succumbed within 12 h and 96% by 24 h, whereas no deaths occurred in the EC-32M group at 12 h and the mortality reached 88% at 24 h (Fig 3H). To further evaluate bacterial dissemination, mice were infected with 5 × 10^7^ CFU of either strain, and bacterial loads in the liver, spleen, lung, and kidney were determined at 12 h post-infection. EC-32M3BB displayed significantly higher bacterial burdens in the liver and spleen, and slightly elevated levels in the lung and kidney, compared with EC-32M. Consistent with these results, an *in vivo* competition assay demonstrated that EC-32M3BB modestly outcompeted EC-32M in the host environment: when mice were co-infected with a 1:1 mixture of the two strains, EC-32M3BB accounted for approximately two-thirds of the total bacterial population in blood samples collected 24 h post-infection, whereas EC-32M comprised only one-third (Fig 3J). Although this competitive advantage was moderate (~2-fold), it supports the notion that enhanced immune escape may allow EC-32M3BB to persist longer *in vivo* and gain an early advantage during infection. Together, these findings indicate that capsule overproduction enhances immune evasion and accelerates host damage, leading to earlier and more severe disease outcomes. While a subtle in-vivo persistence or fitness advantage may coexist, the dominant phenotype appears to be accelerated immune escape-driven pathogenesis rather than a generalized growth advantage.

Importantly, this enhanced pathogenic potential occurred without significant changes in antibiotic susceptibility (S2 Table). Although EC-32M3BB displayed a modestly reduced growth rate compared to EC-32M (Fig 3J), its heightened immune evasion and accelerated lethality highlight the potential risk of capsule-mediated phage resistance, underscoring the need to evaluate phage-resistant variants not only for resistance profiles but also for unintended virulence consequences.

### Emergence of hypermucoid, hypervirulent *E. coli* ST410 strains in clinical settings

Colanic acid biosynthesis in *E. coli* is primarily regulated by the RcsCDB phosphorelay system (Fig 5). Two key regulators, YrfF and Lon, govern this process. YrfF suppresses capsule production by modulating the RcsCDB cascade, whereas Lon controls colanic acid expression by degrading RcsA, a positive regulator within the same pathway [26]. In our resistant isolate EC-32M3BB, YrfF was directly mutated. To explore whether clinical isolates naturally harbor mutations in these key regulatory genes, we analyzed 3,784 publicly available *E. coli* ST410 genomes from NCBI. Mutations in *yrfF* and *lon* against wild type of ST410 were detected in 2.01% (76/3784) and 1.19% (45/3784) of strains, respectively (Fig 3I). Among the 76 *yrfF* variants, one strain carried a large nucleotide insertion, while the remaining 75 strains harbored protein-altering mutations, including 72 missense and 3 nonsense substitutions; in 45 *lon* variants, 44 strains carried missense mutations and 1 carried a nonsense mutation (Fig 3M), suggesting that hypermucoidy may arise through multiple genetic routes targeting the same regulatory network. However, none of these strains carried the *yrfF* T292A mutations identified in this study.

**Fig 4:**
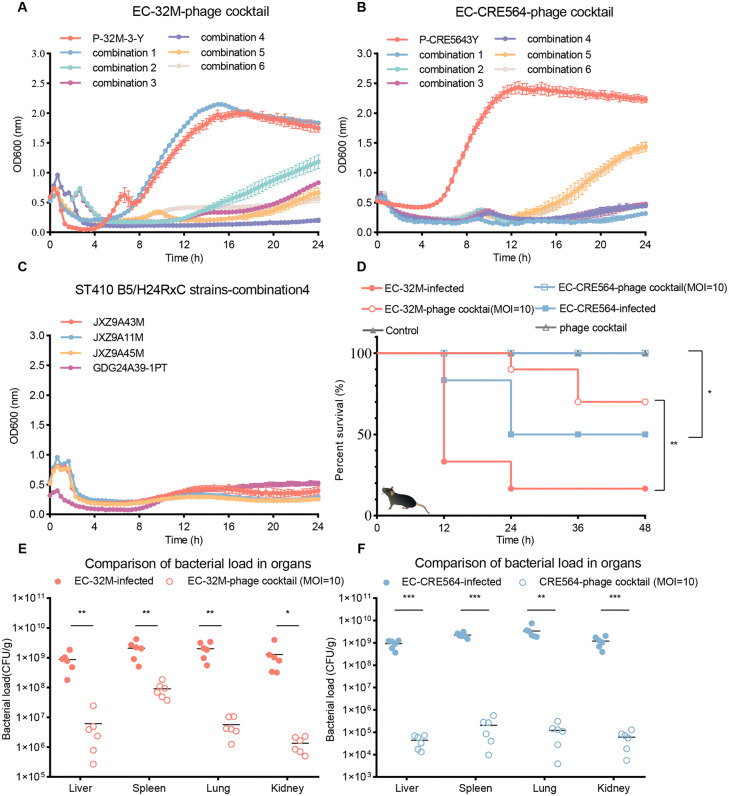
Rational design and therapeutic efficacy of a phage cocktail targeting *E. coli* ST410. **A–B**. Bactericidal activity of different phage cocktail combinations against ST410 strain EC-32M (A) and carbapenem-resistant strain EC-CRE564 (B). **C.** Bactericidal effect of the selected phage cocktail (Combination 4) against additional ST410 B5/H24RxC strains. **D.** Survival curves of mice infected with bacterial strains and treated with phage cocktails. Survival was monitored for 2 days post-infection. Group size: EC-32M-infected (n = 10), EC-32M-phage cocktail (n = 10), EC-CRE564-infected (n = 10), EC-CRE564-phage cocktail (n = 10), Control (n = 6), phage cocktail (n = 6). Statistical analysis was performed using the Log-rank (Mantel–Cox) test. **E–F**. Bacterial burden in major organs (liver, spleen, kidney, lung) of infected mice following phage cocktail treatment: EC-32M-infected mice (E) and EC-CRE564-infected mice **(F)**. Bacterial burden was assessed within 12 hours post-infection, prior to any observed mortality. All samples were collected from viable mice. Group sizes: n = 6 for each group. Values were determined by unpaired two-tailed t-test.

**Fig 5:**
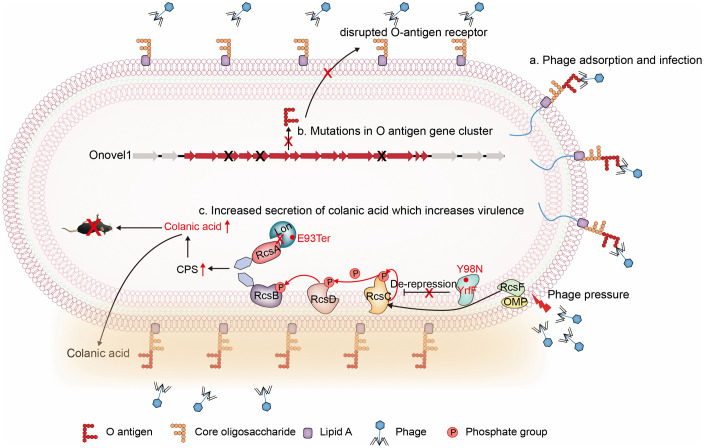
Schematic representation of phage resistance mechanisms in *Escherichia coli* ST410. **a.** Phage receptor recognition: Phage P-32M-3-Y binds to O-antigen on the bacterial outer membrane to initiate infection. **b.** Receptor-mediated resistance: Mutations in the O-antigen biosynthesis cluster disrupt receptor formation, blocking phage adsorption. **c.** Colanic acid–based shielding: Phage pressure selects for a Y98N substitution in YrfF, a negative regulator of the RcsCDB phosphorelay. This inactivation leads to constitutive activation of the Rcs system, triggering overexpression of the cps operon and colanic acid hyperproduction, which forms a protective barrier against phage attack. Furthermore, the E93Ter mutation in the Lon protease could block the degradation of RcsA, which drove unchecked capsule biosynthesis.

Intriguingly, during surveillance of multidrug-resistant pathogens in companion animals, we isolated a naturally mucoid *E. coli* ST410 strain GDG24AEC-39-1PT (hereafter EC-39-1PT) from a hospitalized cat face (Fig 3A). Whole-genome sequencing revealed a G → T transversion in *lon*, introducing a premature stop codon (Glu93Ter). Notably, this Glu93Ter mutation was absent from all 3,784 publicly available ST410 genomes analyzed in this study. Given that Lon protease degrades RcsA, the loss-of-function mutation likely drove unchecked capsule biosynthesis [26]. Complementation with wild-type *lon* fully abolished the mucoid phenotype, reduced colanic acid production and phage sensitivity (Fig 3A, 3C and 3E), confirming its causal role. Functionally, EC-39-1PT exhibited strong resistance to phage P-32M-3-Y and an even greater reduction in macrophage uptake than EC-32M3BB, with ~77-fold lower internalization compared to EC-32M (Fig 3K). In addition to its phagocytosis resistance like EC-32M3BB, EC-39-1PT also showed markedly reduced adhesion to macrophages—approximately half that of both EC-32M and EC-32M3BB (Fig 3J)—further contributing to its enhanced immune evasion. Together, these data reveal the natural emergence of hypermucoid, hypervirulent *E. coli* ST410 strains, likely under selective pressures in clinical environments. The identification of such strains underscores the broader public health threat posed by capsule-mediated resistance and calls for close monitoring in both therapeutic and epidemiological contexts.

### Selection of bacteriophages against phage-resistant *E. coli* ST410 and rational phage cocktail design

To address the rapid emergence of phage-resistant *E. coli* ST410 variants, we performed a secondary phage isolation targeting resistant strains derived from both *in vitro* co-culture and *in vivo i*nfection models. Eight lytic phages were isolated from wastewater and fecal samples and classified into four genera—*Kagunavirus*, *Dhillonvirus*, *Dhakavirus*, and *Phapecoctavirus*—based on whole-genome sequencing and phylogenetic analyses (Table 3). None harbored resistance, virulence, or integrase genes, supporting their therapeutic suitability. Notably, most resistant mutants carried mutations in the O-antigen biosynthesis gene cluster, disrupting phage receptor formation. In addition, the isolate EC-32M3BB acquired *yrfF* mutations that led to colanic acid overproduction and capsule-mediated shielding. These distinct resistance mechanisms informed our cocktail design strategy: selecting phages capable of overcoming both receptor modification and capsule-based evasion, thereby enhancing efficacy against resistant subpopulations.

**Table 3:** The classification of the bacteriophages isolated in the 2nd phage selection study.

Phage ID	Origin	Genome length	Genus	The hypothesized receptor	Reference
P-CRE5643Y	sewage	44,881 bp	*Kagunavirus*	O antigen or K capsular type	[27]
P-S15S	sewage	169,505 bp	*Dhakavirus*	FadL	[28]
P-S17S	Stool	169,396 bp	*Dhakavirus*	FadL	[28]
P-3BB12S	Stool	150,584 bp	*Phapecoctavirus*	Enterobacter common antigen (ECA) and outer LPS core first glucose	[29]
P-3BB5Y	sewage	151,853 bp	*Phapecoctavirus*	Enterobacter common antigen (ECA) and outer LPS core first glucose	[29]
P-3BB6Y	Stool	146,712 bp	*Phapecoctavirus*	Enterobacter common antigen (ECA) and outer LPS core first glucose	[29]
P-3BB7Y	Stool	146,711 bp	*Phapecoctavirus*	Enterobacter common antigen (ECA) and outer LPS core first glucose	[29]
P-S15B	sewage	44,780 bp	*Dhillonvirus*	LptD	[30]

Phylogenetic analysis revealed distinct receptor usage patterns, predicting P-CRE5643Y to target the O-antigen or K capsule, P-S17S to use FadL, P-S15B to bind LptD, and P-3BB6Y to recognize ECA or LPS core glucose; notably, P-3BB6Y also encodes a colanic acid-degrading protein (S6 Fig). TEM imaging revealed predominantly *Myoviridae* morphologies (6/8), with two *Siphoviridae* phages (2/8) (S8 Fig). Host range profiling showed that 7 of 8 phages lysed diverse phage-resistant ST410 isolates (S3 Fig).

Building on these findings, we designed a rational phage cocktail comprising nine phages, including P-32M-3-Y and P-CRE5643Y to target the primary ST410 hosts, EC-32M (pet-derived) and GDG23ZDCRE564 (human-derived, hereafter EC-CRE564), and one representative phage from each genus to target resistant variants and maximize receptor coverage (S3 Table). Killing assays (MOI = 10) identified combination 4 (P-32M-3-Y, P-CRE5643Y, P-3BB6Y, P-S17S) as the most effective, achieving sustained bacterial suppression (Fig 4A, 4B). This cocktail also lysed additional B5/H24RxC clinical isolates including the mucoid strain EC-39-1PT (Fig 4C).

In murine peritoneal infection models, phage therapy fully protected against EC-CRE564 infection (100% survival) and significantly improved survival in EC-32M-infected mice (70% survival vs 15% in untreated controls) (Fig 4D). Moreover, phage treatment markedly reduced bacterial burdens in the liver, spleen, lung, and kidney against the EC-32M and EC-CRE564 infections (Fig 4E, 4F). These results demonstrate the feasibility of rational phage cocktail design to overcome resistance and enhance therapeutic outcomes against multidrug-resistant ST410 strains.

## Discussion

Interactions between bacteriophages and bacteria are shaped by an ongoing evolutionary arms race, frequently resulting in fitness trade-offs such as increased antibiotic susceptibility or reduced virulence. Surprisingly, in this study, screening against the high-risk *E. coli* ST410 lineage uncovered a phenotype in which phage resistance was accompanied by an increased virulence potential, likely driven by enhanced immune evasion.

Capsular polysaccharides, forming the outermost layer of bacterial cells, are major virulence factors that protect pathogens from host immune clearance [28,31,32]. Previous studies have shown that capsule-mediated phage resistance, such as in *E. coli* K15, can preserve bacterial virulence without enhancement [33]. In contrast, our data reveal that YrfF mutation-driven hyperproduction of colanic acid confers both increased phage resistance and elevated pathogenicity. We propose that this divergence may stem from differences in capsule composition; as observed in *Klebsiella pneumoniae*, capsular structure and its interaction with host immunity can significantly influence virulence outcomes [34].

Colanic acid, composed of repeating units of glucose, galactose, fucose, and glucuronic acid [35], plays a central role in protecting bacteria against environmental stresses such as oxidative damage, acidity, and osmotic pressure [36]. Beyond stress protection, colanic acid has been implicated in enhancing *E. coli* O157:H7 attachment to plant surfaces, potentially facilitating foodborne transmission [37], and in shielding extraintestinal pathogenic *E. coli* (ExPEC) from serum-mediated killing [38]. Our findings extend these roles, demonstrating that colanic acid not only enables phage evasion but also amplifies bacterial virulence. To our knowledge, this is the first evidence that phage resistance can actively potentiate virulence, highlighting an underappreciated risk in the therapeutic application of phages.

In *Enterobacteriaceae*, colanic acid biosynthesis is regulated by the conserved RcsCDB phosphorelay [26,39,40]. Outer membrane perturbations trigger RcsF-mediated activation of RcsC, with signal transduction through RcsD to RcsB; the RcsB-RcsA heterodimer subsequently activates the cps operon, driving colanic acid overproduction (Fig 5). Under non-stress conditions, YrfF represses this pathway by directly inhibiting RcsC. Capsule-mediated resistance, unlike receptor mutations, provides broad-spectrum, non-specific protection against diverse phages [33,41]. Consistently, both the EC-32M3BB strain and BW25113 carrying the YrfF Y98N mutation exhibited robust resistance not only to P-32M-3-Y but also to multiple unrelated phages. Similarly, CRISPRi silencing of IgaA (a YrfF homolog) induced colanic acid overproduction and multi-phage resistance [42], underscoring the conserved role of the Rcs system in phage defense.

We also identified a hypermucoid ST410 isolate from a companion animal carrying a *lon* mutation, impairing RcsA degradation. Similar mutations in negative Rcs regulators are present in public genome datasets. While the evolutionary pressures remain unclear, our findings suggest that phage-driven selection may favor colanic acid hyperproduction. Given its dual role in resistance and virulence, the mucoid phenotype may represent an adaptive strategy in *E. coli* ST410 and broader *Enterobacteriaceae* populations, warranting further investigation.

Beyond colanic acid-mediated resistance, our findings identify O-antigen modification as the primary phage evasion strategy in *E. coli* ST410, consistent with previous reports that receptor alteration is the predominant mechanism of phage resistance [43]. Notably, O-antigen mutations emerged under both *in vitro* and *in vivo* phage selection, suggesting that *in vitro* resistance patterns may partially anticipate resistance mechanisms arising during therapeutic application [33]. The O antigen, a highly variable surface structure encompassing over 180 known *E. coli* serogroups [44], can differ by as little as a single sugar residue (e.g., O107 vs. O117) [45]. Genetic alterations, including point mutations and insertions, can disrupt or modify O-antigen biosynthesis, altering phage susceptibility [45–48]. The extensive O-antigen diversity among ST410 subclones, together with phage-driven antigenic shifts observed in this study, underscores phage predation as a powerful yet underappreciated driver of surface antigen diversification [49]. Given that O antigens are frequent targets for bacterial vaccines, their extensive variability poses a significant challenge for effective vaccine design [50]. These findings highlight the need for more comprehensive therapeutic strategies.

To mitigate therapeutic resistance, we employed an iterative phage screening strategy targeting both *in vitro*-derived resistant mutants and clinical isolates. Consistent with previous reports [51], our multi-phage cocktail exhibited enhanced efficacy against hypervirulent, carbapenem-resistant B5/H24RxC subclones compared to monophage treatment. However, the cocktail did not achieve complete coverage of all ST410 variants, highlighting the potential need for adjunctive antibiotic therapy to broaden therapeutic outcomes. While *in vivo* validation in clinical settings is lacking, our results demonstrate the promise of phage cocktails for treating *E. coli* ST410 infections in both companion animals and humans. Future studies should also explore additional, uncharacterized resistance mechanisms that may emerge during long-term phage-bacteria coevolution.

In conclusion, our findings highlight the dynamic interplay between O-antigen modification and phage evasion as central drivers of the ongoing bacteria–phage evolutionary arms race. Critically, we demonstrate that colanic acid–mediated phage resistance is associated with enhanced virulence potential, primarily through increased immune-escape capacity and accelerated disease progression *in vivo*. Given that *E. coli* ST410 harbors a high-pathogenicity island, the acquisition of a hypermucoid phenotype via colanic acid overproduction may further intensify disease severity in an already pathogenic background. Given the reduced growth fitness observed *in vitro*, the phenotypic outcome observed here appears to be driven predominantly by enhanced immune evasion rather than growth-related advantages. The identification of a hypermucoid, hypervirulent ST410 clinical isolate further suggests that capsule-mediated adaptations may be more widespread in natural populations than previously appreciated. This observation raises concern that capsule-mediated phage resistance in high-risk lineages may increase virulence, representing an underrecognized threat in clinical and veterinary contexts. Monitoring capsule-associated evolutionary trajectories and evaluating the biological risks of capsule-based resistance will be essential for safe and effective phage therapy, particularly against emerging hypermucoid and hypervirulent pathogens.

## Meterials and methods

### Ethics statement

All animal experiments were approved by the Animal Protection and Utilisation Committee of South China Agricultural University, Guangzhou, China (APUC No. 2024c028 and APUC No. 2025c126), and were conducted in accordance with national ethical guidelines for the care and use of laboratory animals.

### Bacterial strains for phage isolation

The *E. coli* ST410 strain JXZ9A32M (EC-32M), recovered on MacConkey agar plates (Hopebio, Qingdao, China) supplemented with 0.5 μg/mL meropenem from a canine stool sample at a pet hospital in Jiangxi Province, was used as the host for phage isolation.

The *E. coli* ST410 strain GDG24AEC-39-1PT (EC-39-1PT), recovered on MacConkey agar plates (Hopebio, Qingdao, China) supplemented with 4 μg/mL tigecycline from a cat stool sample at a pet hospital in Guangdong Province.

The *E. coli* ST410 strain GDG24ZDCRE564 was isolated from the feces of a female patient with acute lymphoblastic leukemia during a CRE (carbapenem-resistant *Enterobacteriaceae*) screening at a hospital in Guangzhou.

The three strains underwent whole-genome sequencing, with JXZ9A32M and GDG24AEC-39-1PT further subjected to long-read sequencing.

### Phage isolation and purification

Untreated wastewater was collected from a slaughterhouse in Panyu District, Guangzhou. Phage isolation was performed with minor modifications from a previously described method [52]. Samples were pre-filtered through sterile gauze to remove debris, centrifuged at 4,000 × *g* for 20 min at 4 °C, and sequentially filtered through 0.45 μm and 0.22 μm membranes (Jinteng, Tianjin, China) to remove bacteria. The filtrate was concentrated to ~50 mL using tangential flow filtration (Millipore, USA). For phage enrichment, 10 mL of the concentrated filtrate was mixed with 10 mL of 2 × LB broth (Hopebio, Qingdao, China) containing 2 mM CaCl₂, inoculated with 100 μL of overnight *E. coli* EC-32M culture, and incubated at 37°C with gentle shaking (50 rpm) for 24–48 h. After centrifugation at 10,000 × *g* for 10 min, the supernatant was filtered through a 0.22 μm membrane.

Phages were isolated by serial dilution of the filtrate in SM buffer (2 × ; Sangon Biotech, Shanghai, China). Next, the filtrate containing enriched phages (0.1 ml) was mixed with 0.1 ml of the host cells (OD_600 _= 0.4-0.9) in LB medium and 5 ml of molten top soft nutrient agar (0.7%). The mixture was overlaid onto solidified base nutrient agar (1.5%). Phages were purified according to the standard methods [53]. Phage titers were determined with the double-layered method. The isolated bacteriophages were stored in 50% glycerol at -80°C. Using the same protocol, three additional lytic phages were independently isolated.

To assess the therapeutic potential of phage P-32M-3-Y, its host range was determined against a panel of additional *E. coli* strains (Table 1 and S1 Table) using the spot assay as previously described [54]. The panel included *E. coli* strains representing twelve sequence types (ST57, ST156, ST167, ST195, ST206, ST410, ST746, ST761, ST1011, ST9124, and ST9388) to evaluate cross-ST lytic activity [15,16,55–57]. To minimize false positives, assays were performed using high-titer phage stocks (~10⁸ PFU/mL). Bacterial sensitivity to a given bacteriophage was evaluated in the basis of the lysis-cleared zone at a spot: bacteria were differentiated into two categories according to the clarity of plaques: lysis (+) and no lysis (-).

### Multiplicity of infection (MOI) assay

To determine the optimal MOI for maximal phage progeny production, the host strain EC-32M was grown to logarithmic phase and adjusted to 10⁸ CFU/mL. One milliliter of bacterial culture was mixed with 10 μL of serial 10-fold diluted phage suspensions (ranging from 10² to 10⁹ PFU/mL). After 4 h incubation at 37°C, progeny phage titers were quantified using the double-layer agar method [58].

### One-step growth curve

A one-step growth assay was performed to estimate the latent period and burst size of phage P-32M-3-Y as previously described with minor modifications [59]. Briefly, 100 μL of phage suspension (10⁵ PFU/mL) was mixed with 1 mL of host bacteria (10⁸ CFU/mL) in 8.9 mL LB broth and allowed to adsorb at 37°C for 10 min. The mixture was centrifuged at 5,000 rpm for 3 min, and free phages in the supernatant were quantified by filtration (0.22 μm). The pellet was resuspended in fresh LB and incubated at 37°C with shaking (180 rpm), and samples were taken every 10 min for phage titration. Burst size was calculated as the ratio of final phage count to the number of initially infected cells. All assays were performed in triplicate.

### Phage adsorption assay and relative adsorption efficiency

Phage adsorption kinetics were evaluated by mixing 10 mL of host culture (10⁸ CFU/mL) with 100 μL of phage suspension (10⁸ PFU/mL) at an MOI of 0.01. Samples were collected at 1, 3, 5, 7, 10, 13, 16, 20, and 25 min, centrifuged (8,000 × g, 1 min), and filtered (0.22 μm) to determine the concentration of unadsorbed phages. Relative adsorption efficiency to different strains was assessed at 10 min post-infection using the formula: Relative adsorption rate = (Initial titer−Residual titer in test strain)/ (Initial titer−Residual titer in control strain) × 100%.

### Phage stability under different pH and temperature conditions

To assess pH stability, 100 μL of phage lysate (5 × 10⁹ PFU/mL) was incubated with 900 μL of SM buffer adjusted to pH 3–13 at 25°C for 1 h. For thermal stability, phage suspensions were incubated at 4, 25, 37, 50, 60, and 70°C for 1 h in a water bath. Phage titers were determined using the double-layer agar method [58]. All experiments were performed in triplicate.

### Phage lysis curve

To evaluate lytic activity, mid-log-phase *E. coli* cultures (OD₆₀₀ = 0.6) were mixed 1:1 with phage lysates at MOIs of 10, 1, 0.1, and 0.01. LB-only cultures served as negative controls. Cultures were incubated statically at 37°C, and OD₆₀₀ readings were recorded hourly for 12 h using a microplate reader (PE Ensight, Revvity, USA). Each condition was tested in biological triplicates.

### Transmission electron microscopy (TEM)

Phage morphology was examined using TEM. Phage particles were concentrated using a 100 kDa ultrafiltration device (Millipore, USA), deposited onto copper grids for 15 min at room temperature, and negatively stained with 1.0% phosphotungstic acid (pH 7.0) for 10 min. After drying, the grids were visualized using a transmission electron microscope (FEI Talos L120C, Czech Republic) at an accelerating voltage of 80 kV.

### Whole-genome sequencing and analysis

Whole-genome sequencing was performed to classify phages and elucidate resistance mechanisms in *E. coli* JXZ9A32M mutants. Phage genomic DNA was extracted using the Omega viral DNA kit (Omega Bio-Tek, USA), and bacterial genomic DNA was isolated using the Hipure Bacterial DNA Kit (Magen, Guangzhou, China). DNA was fragmented to ~350 bp and used to construct 150 bp paired-end libraries for sequencing on the Illumina NovaSeq 6000 platform (Illumina, USA). The long-read sequencing was performed using the Oxford Nanopore MinION platform (Oxford Nanopore Technologies, UK).

Reads were assembled using SPAdes v3.8.7 (Illumina) and Unicycler v0.4.7 (Nanopore). For phages, circular contigs were identified from assembly.graph.fastg files using Bandage and extracted as complete genomes. Nanopore assemblies were polished with Unicycler to generate high-quality consensus sequences.

Phage genomes were annotated using the RAST server (http://rast.nmpdr.org/) and validated via BLASTP against the NCBI RefSeq database. Genome maps and GC-skew were visualized using CGView (https://cgview.ca/). Biosafety profiling included virulence factor screening (VFDB, http://www.mgc.ac.cn/VFs/), antibiotic resistance gene detection (CARD, https://card.mcmaster.ca/), and tRNA prediction (tRNAscan-SE 2.0, https://card.mcmaster.ca/).

Phage tail fiber proteins were compared using BLASTP, and homologous sequences were aligned and visualized with Easyfig v2.2.5. *E. coli* serotypes were predicted using SerotypeFinder 2.0. For O-nontypeable strains, conserved O-antigen flanking regions were identified via BLASTn and visualized with Easyfig.

### Bacterial growth curve

Overnight cultures were prepared by inoculating single colonies into 2 mL LB broth and incubating at 37°C with shaking (180 rpm). Cultures were diluted to an initial OD₆₀₀ of 0.01 in 50 mL fresh LB, and growth was monitored hourly by measuring OD₆₀₀ (200 μL per well in 96-well plates; LB as blank) using a microplate reader (Ningbo Scientz, China). Strain was tested in biological triplicates under identical conditions.

### Antimicrobial susceptibility testing

Based on the Clinical and Laboratory Standards Institute (CLSI) guidelines (M07-A11), antimicrobial susceptibilities of isolates against 10 antibiotics were tested: cefotaxime (CTX), imipenem (IMP), ciprofloxacin (CIP), gentamicin (GEN), amikacin (AMK), fosfomycin (FOS), tigecycline (TIG), doxycycline (DOX), florfenicol (FFC), and colistin (CL). The agar dilution method was used, except for colistin and tigecycline, got which broth microdilution was used; the quality control was *E. coli* ATCC 25922. The results were interpreted based on the criteria in CLSI (M100-S30) and EUCAST (http://www.eucast.org/clinical_breakpoints/).

### Construction of plasmids and strains

Primers used in this study are listed in S4 Table. PCR amplification was performed with 2 × Phanta Flash Master Mix (Vazyme Biotech, China). The *yrfF* gene was amplified from *E. coli* ST410 JXZ9A32M genomic DNA using primers yrfF-pBAD-F/R. To replace the ampicillin resistance gene in pBAD24, a chloramphenicol acetyltransferase (cat) cassette was amplified from pHSG575-cm using primers cm-F/R, based on the antibiotic profile of strain EC-32M3BB (ampicillin-resistant, chloramphenicol-sensitive). The pBAD24 backbone was modified by amplifying the arabinose-inducible promoter (primers pBAD24-F/R) and deleting the *bla* gene (primers pBAD-KO-AMP-F/R). Constructs were assembled using a seamless cloning kit (Genesand Biotech, China) and served as templates for homologous recombination.

Electrocompetent *E. coli* EC-32M3BB cells were prepared via repeated washing with 10% glycerol at 4°C. Plasmids (pBAD24-yrfF, pBAD24-cm) were introduced by electroporation (1 mm cuvettes, 1.8 kV; Bio-Rad, USA), followed by recovery in LB (37°C, 1 h) and selection on LB agar with 30 μg/mL chloramphenicol. The plasmid pBAD24-lon was constructed similarly, using primers listed in S4 Table.

The yrfF Y98N point mutation was introduced into *E. coli* BW25113 using a CRISPR-Cas9 system as previously described [60]. The PAM sequence used was acacacgctatcacgcgtta. All constructs were validated by PCR and Sanger sequencing.

### Transcriptomic analysis, RNA extraction, and RT-qPCR

Biological triplicates of *E. coli* ST410 strains (EC-32M and EC-32M3BB) were grown in LB broth (37°C, 180 rpm) to mid-log phase (OD₆₀₀ = 0.6). Bacterial pellets were harvested by centrifugation (5,000 × *g*, 10 min), washed twice with PBS, and flash-frozen in liquid nitrogen. RNA sequencing was performed by Novogene (China). Raw reads were mapped to the most closely related *E. coli* genomes available in the NCBI database. Gene expression levels were normalized using DESeq2 v1.20.0, and differentially expressed genes (DEGs) were identified based on an adjusted *p* < 0.05 and |log₂FC| > 1. Volcano plots were generated with ggplot2 v3.4.2, and GO/KEGG pathway enrichment was conducted using clusterProfiler v3.8.1 on the NovoCloud platform.

For RT-qPCR, total RNA was extracted from equal numbers of cells (mid-log phase) using the E.Z.N.A. Bacterial RNA Kit (Omega Bio-Tek, USA) following the manufacturer’s instructions. RNA integrity was assessed by 1% agarose gel electrophoresis, and purity/concentration were measured with a NanoDrop 2000c (Thermo Fisher Scientific, USA). Samples with 23S/16S rRNA ratios of ~2.0 and A260/A280 ratios between 1.8–2.2 were used for downstream applications. cDNA was synthesized from ~1 μg total RNA using HiScript III All-in-One RT SuperMix Perfect for qPCR (Vazyme Biotech, China).

Quantitative PCR was performed using 2 × ChamQ Universal SYBR qPCR Master Mix (Vazyme Biotech) on a 96-well real-time PCR system (Thermo Fisher Scientific). Reactions (20 μL) contained 10 μL SYBR mix, 1 μL diluted cDNA (1:10), 0.4 μL of each primer (10 μM), and 8.2 μL ddH₂O. Cycling conditions were: 95°C for 30 s; 40 cycles of 95°C for 10 s and 60°C for 30 s; followed by melting curve analysis (65–95°C). Expression levels were normalized to the 16S rRNA gene, and relative gene expression levels were calculated using the 2^⁻ΔΔCt^ method [61].

### Colanic acid extraction and quantification

The method used to extract colanic acid was based on the procedure previously described with minor modifications [62]. The strains was streaked onto LB agar plates and incubated at 37 °C for 12 hours. Colonies were scraped into 1 mL of sterile distilled water, and the suspension was adjusted to an OD_600_ of 4. The cell cultures were heated for 20 min at 100 ℃ to denature EPS-degrading enzymes. After cooling, they were centrifuged at 16,000g for 20min. Next, the supernatant was collected and diluted 10-fold to fit within the standard curve. 111 μL of the diluted mixture was added to 500 μL H_2_SO_4_/Water (6:1 v/v) and the mixture was heated to 95 °C for 30 min then cooled to room temperature. For each sample, to a well of a flat-bottomed 96-well place was added (a) 5 μL cysteine hydrochloride (Cys·HCl, 3% stock solution) or (b) ddH_2_O. To each of these was added 200 μL of the cooled acidified EPS mixture. The absorbance of both (a) and (b) was measured at 396 and 427nm. The absorbance measurements at both 396 and 427nm without Cys·HCl were subtracted from those with Cys·HCl to provide background corrected A_396_ and A_427_ values. The final absorbance values were calculated by subtracting A_427_ from A_396_. The result was directly correlated to fucose concentration by using a fucose standard curve ranging from 5 μg/mL to 100 μg/mL. A representative standard curve is y = 0.0026x + 0.0197 (y: A_396_-A_427_, x: L-fucose concentration (μg/ mL), R^2^ = 0.9947. Statistical comparisons of relative proportions between strains were performed using an unpaired two-tailed Student’s *t*-test (GraphPad Prism 9), with *P* < 0.05 considered significant. Results are presented as mean ± SD.

### Macrophage adhesion and phagocytosis assay

The culture of RAW264.7 cells, the adhesion assay and the anti-phagocytosis assay were performed with minor modifications based on previously reported methods [63,64]. The RAW264.7 cell line was cultured in Dulbecco’s Modified Eagle Medium (DMEM, Gibco, Waltham, MA, USA) supplemented with 10% fetal bovine serum (FBS, Procell, China).

For adhesion assay, RAW264.7 cells were seeded at 2 × 10⁵ cells per well in 12-well plates and infected with *E. coli* strains EC-32M, EC-32M3BB, or GDG24AEC-39-1PT at a multiplicity of infection (MOI) of 10 for 1.5 h. Then, monolayers were washed 3 times using PBS buffer and disrupted with 0.1% Triton X-100. Serial dilutions were performed and plated. The adhesion index was calculated as CFU/mL 90 min p.i. divided by CFU/mL in the inoculum.

For phagocytosis assay, RAW264.7 cells were seeded at 2 × 10⁵ cells per well in 12-well plates and infected with *E. coli* strains EC-32M, EC-32M3BB, or GDG24AEC-39-1PT at a multiplicity of infection (MOI) of 10 for 2 h. To eliminate extracellular bacteria, cells were incubated for 1.5 h in culture medium containing 150 μg/mL apramycin, a membrane-impermeable antibiotic. Cells were then washed with PBS and lysed with 0.1% Triton X-100 for 10 min to release intracellular bacteria. The number of internalized bacteria was determined by plating serial dilutions. Invasion inhibition rates were calculated as: [(CFU of infected control group−CFU of experimental treatment group)/ CFU of infected control group]×100%. All assays were performed in triplicate.

### Mouse virulence experiment and bacterial load experiment

Five-week-old female C57BL/6 mice were obtained from Zhuhai Bestest Company and housed in a specific-pathogen-free (SPF) facility under controlled conditions (25 ± 2 ⁰C, 50% relative humidity, 12 h light/dark cycle) with a one-week acclimatization period prior to experiments. For the single bacteriophage treatment assay (Fig 1H), mice were intraperitoneally injected with 1 × 10⁸ CFU of strain EC-32M. Phage treatment with 1 × 10^9^ PFU was administered intraperitoneally 4 h post-infection. The EC-32M-infecetd group and the control group were administered intraperitoneally with PBS at the same time. Group size: EC-32M-infected (n = 8), EC-32M-P-32M-3-Y(MOI = 10) (n = 10), Control (n = 6). For statistical analysis, the Gehan–Breslow–Wilcoxon test was applied.

For survival analysis (Fig 3H), mice were intraperitoneally injected with 5 × 10^7^ CFU of either strain EC-32M (n = 25) or EC-32M3BB (n = 25). The control group were administered intraperitoneally with PBS (n = 6) at the same time. Survival was monitored at 12, 24, 48, and 72h post-infection. To assess bacterial burden, mice were intraperitoneally injected with 5 × 10^7^ CFU of either strain EC-32M (n = 6) or EC-32M3BB (n = 6). Then, mice were euthanized and tissues (liver, spleen, lung, and kidney) were aseptically collected within 12 hours post-infection. At this time point, no mortality had occurred, and all samples were obtained from viable animals to ensure consistency in disease stage. Organs were homogenized in 1 mL sterile PBS at 4 °C using a Tissuelyser-24 homogenizer (Jingxin, Shanghai, China; 60 Hz, 2 min), and the homogenates were serially diluted. Aliquots (50 μL) were plated on LB agar and incubated at 37 °C for 18 hours for colony enumeration. Bacterial burden data were compared between groups using an unpaired two-tailed Student’s *t*-test.

### Mouse *in vivo* competition assay

For *in vivo* competion assay (Fig 3I), the wild-type strain EC-32M and EC-32M3BB were grown in LB broth at 37°C with shaking. Cultures were washed twice with sterile PBS, and adjusted to an optical density corresponding to ~5 × 10^8^ CFU/mL for each strain. Equal volumes of the two bacterial suspensions were mixed to achieve a 1:1 ratio, and each mouse was inoculated intraperitoneally with 100 μL of the mixture (final dose: 2.5 × 10^7^ CFU per strain per mouse). The input ratio was calculated as the formula below with viriation <0.1 log.


$$\text{input}\;\text{ratio}=\frac{\text{CFU}(\text{EC}-\text{32M})}{\text{CFU}(\text{EC}-\text{32M3BB})}$$

At 24 h post-infection, 5 µL of blood was collected from the tail vein of each mouse into heparinized tubes with 200 μL PBS. Blood samples were serially diluted in sterile PBS and plated on n LB agar with 0.5 μg/mL meropenem for total CFU enumeration. To discriminate EC-32M from EC-32M3BB, individual colonies were picked and subjected to colony PCR targeting the *yrfF* locus; PCR products were Sanger-sequenced to confirm strain identity. Primers used were: *yrfF*-F and *yrfF*-R (S4 Table). The relative abundance of each strain was calculated as:


$$\text{Relative}\;\text{proportion}\;\text{of}\;\text{strain}\;\text{X}=\frac{\text{CFU}(\text{X})}{\text{CFU}(\text{EC}-\text{32M})+\text{CFU}(\text{EC}-\text{32M3BB})}$$

Statistical comparisons of relative proportions between strains were performed using an unpaired two-tailed Student’s *t*-test (GraphPad Prism 9), with *P* < 0.05 considered significant. Results are presented as mean ± SD.

### Murine survival and bacterial burden assays following phage cocktail treatment

Mice were intraperitoneally inoculated with either *E. coli* strain EC-32M or EC-CRE564 (1 × 10^8^ CFU). Four hours post-infection, mice received an intraperitoneal injection of a four-phage cocktail (P-32M-3-Y, P-CRE5643Y, P-3BB6Y, P-S17S, each at 1 × 10^9^ PFU, total 4 × 10^9^ PFU) or an equal volume of PBS buffer as control. For survival assays, mice were monitored every 12h for 2 days (Fig 4D). Group size:EC-32M-infected (n = 10), EC-32M-phage cocktail (n = 10), EC-CRE564-infected (n = 10), EC-CRE564-phage cocktail (n = 10), phage cocktail (n = 6), control (n = 6). Survival curves were analyzed using the Log-rank (Mantel–Cox) test.

For bacterial burden assays (Fig 4E and 4F), mice were euthanized at 12 h post-infection, and the liver, spleen, lungs, and kidneys were aseptically collected (n = 6 per group). At this time point, no mortality had occurred, and all samples were obtained from viable animals to ensure consistency in disease stage. Organs were homogenized in 1 mL sterile PBS at 4 °C using a Tissuelyser-24 homogenizer (Jingxin, Shanghai, China; 60 Hz, 2 min), and the homogenates were serially diluted. Aliquots (50 μL) were plated on LB agar and incubated at 37 °C for 18 hours for colony enumeration. Bacterial burden data were compared between groups using an unpaired two-tailed Student’s *t*-test.

### Data visualization

Materials for drawing (Figs 1H, 3H, 4D, and 5) were downloaded from Wikimedia Commons (https://commons.wikimedia.org). The plots were created using GraphPad Prism 9.

## Supporting information

S1 Fig The morphology of phage-resistant bacterial strains. A. Emergence of phage-resistant colonies following high-MOI co-culture of *E. coli* EC-32M with phage P-32M-3-Y. Individual colonies of varying sizes appeared on the plate within 8 hours, indicating rapid development of phage resistance. B. The morphology of O antigen synthesis genes mutation mediated-phage resistant bacterial strains.
(TIF)

S2 Fig Transcriptome expression analysis. A. Volcano plot of differential expression analysis (horizontal axis: log_2_FoldChange values; vertical axis: -log_10padj_). B. KEGG enrichment analysis (horizontal axis: ratio of genes annotated to the function to total differential genes; vertical axis: enriched pathways. Scatter plot uses dots of different colors and sizes, with redder colors indicating more significant enrichment and larger dots representing more enriched genes. The red box highlights significantly enriched pathways). C. Fold change of upregulation in capsule polysaccharide biosynthesis-related genes in the transcriptome.
(TIF)

S3 Fig The host range of the phages isolated in the second round of screening against ST410 strains and resistant bacteria generated in *vitro* and in *vivo.* Red and white blocks represent with and without lysis effect, respectively.
(TIF)

S4 Fig Gene map of the phage P-32M-3-Y. Different colored arrows represent predicted CDSs coding different functions: purple, structure module; yellow, regulation module; blue, packaging module; red, replication module; light green, lysis module; dark green, tRNA; grey, hypothetical protein. The genome map was generated using CGview Server.
(TIF)

S5 Fig Gene map of the phage P-CRE5643Y. (TIF)

S6 Fig Gene map of the phage P-3BB6Y. (TIF)

S7 Fig Gene map of the phage P-S17S. (TIF)

S8 Fig Transmission electron micrographs of the 8 phages. (TIF)

S1 Table The host range of phage P-32M-3-Y against other ST *Escherichia coli.* *** ***+ , P-32M-3-Y could form a clear zone or plaque; -, P-32M-3-Y could not form a clear zone or plaque.
(DOCX)

S2 Table Changes in antibiotic susceptibility of the phage-resistant strain EC-32M3BB. (DOCX)

S3 Table Composition of phage cocktails used in Fig 4A and 4B. Cocktails were designed primarily based on differences in phage receptor usage to broaden host range coverage. The combinations shown represent a subset of possible designs.
(DOCX)

S4 Table The primers used in this study. (DOCX)

S1 Data Original data from animal experiments. (ZIP)
