# Supplementary figures and images for: Colanic acid-mediated phage resistance enhances virulence in high-risk global clone Escherichia coli ST410
Source: PLoS Pathog. 2025 Dec 22;21(12):e1013807. doi: 10.1371/journal.ppat.1013807 (PMC12753057; doi:10.1371/journal.ppat.1013807)

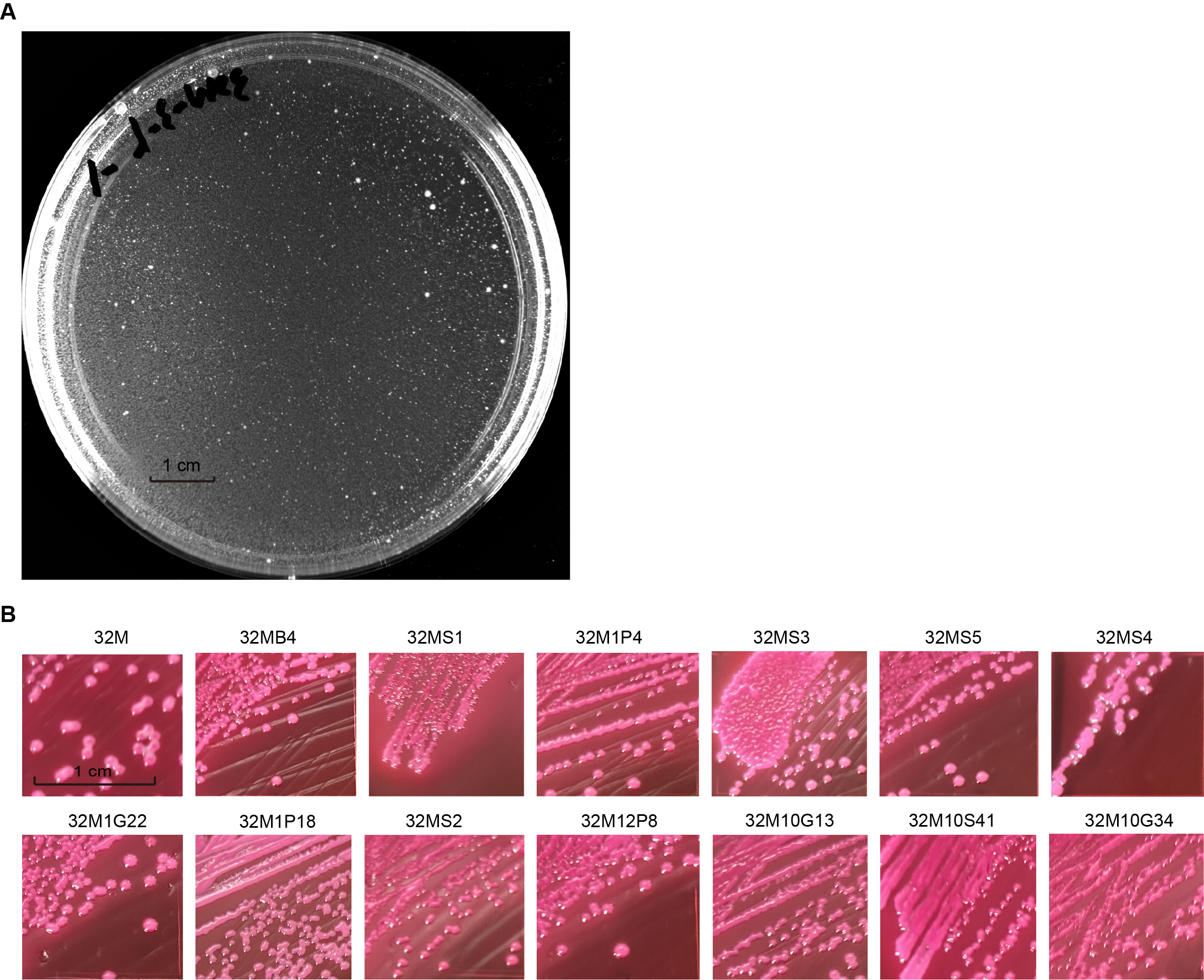

Supplement: S1 Fig — A. Emergence of phage-resistant colonies following high-MOI co-culture of E. coli EC-32M with phage P-32M-3-Y. Individual colonies of varying sizes appeared on the plate within 8 hours, indicating rapid development of phage resistance. B. The morphology of O antigen synthesis genes mutation mediated-phage resistant bacterial strains. (TIF) [file ppat.1013807.s001.tif]

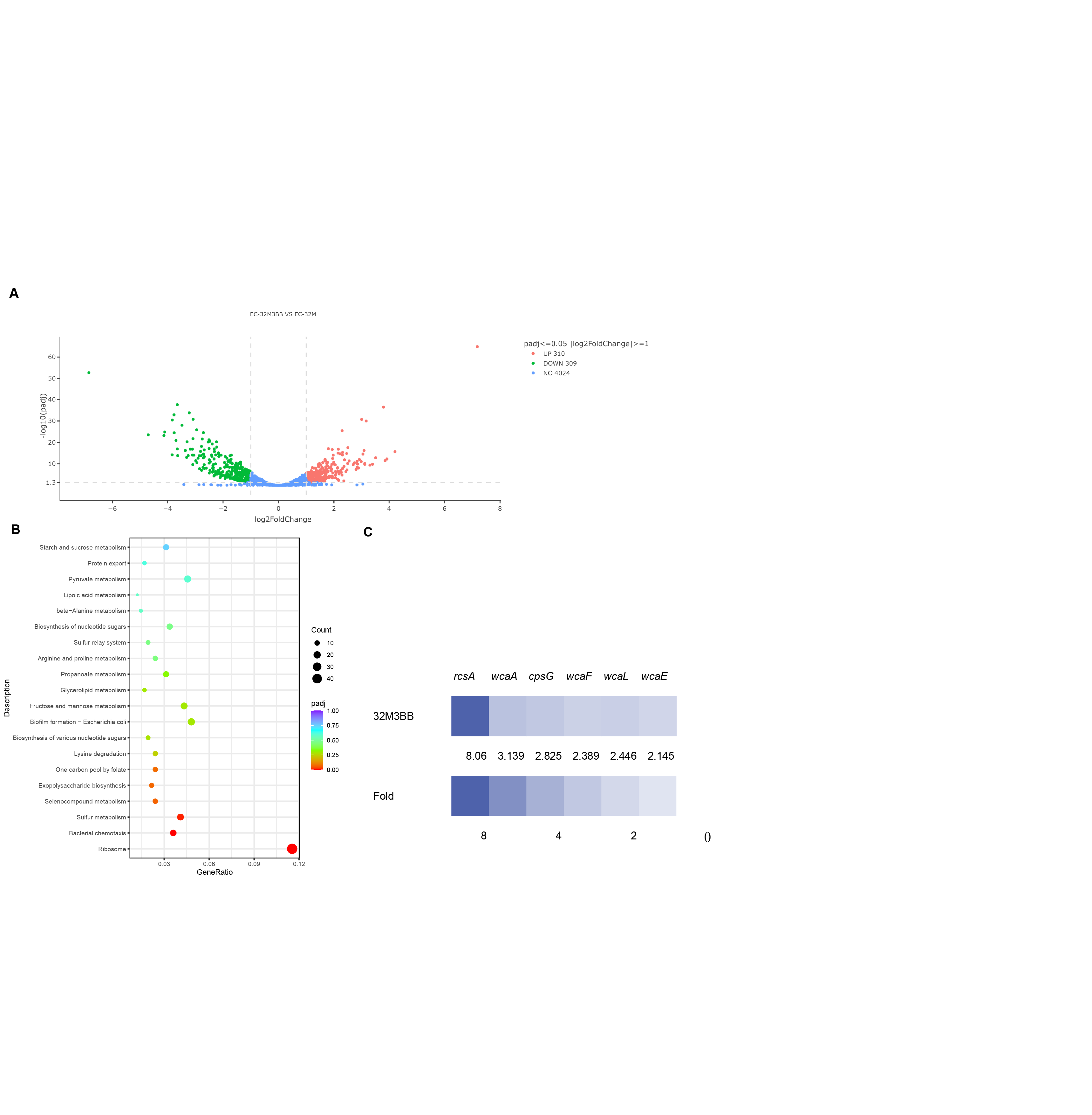

Supplement: S2 Fig — A. Volcano plot of differential expression analysis (horizontal axis: log2FoldChange values; vertical axis: -log10padj). B. KEGG enrichment analysis (horizontal axis: ratio of genes annotated to the function to total differential genes; vertical axis: enriched pathways. Scatter plot uses dots of different colors and sizes, with redder colors indicating more significant enrichment and larger dots representing more enriched genes. The red box highlights significantly enriched pathways). C. Fold change of upregulation in capsule polysaccharide biosynthesis-related genes in the transcriptome. (TIF) [file ppat.1013807.s002.tif]

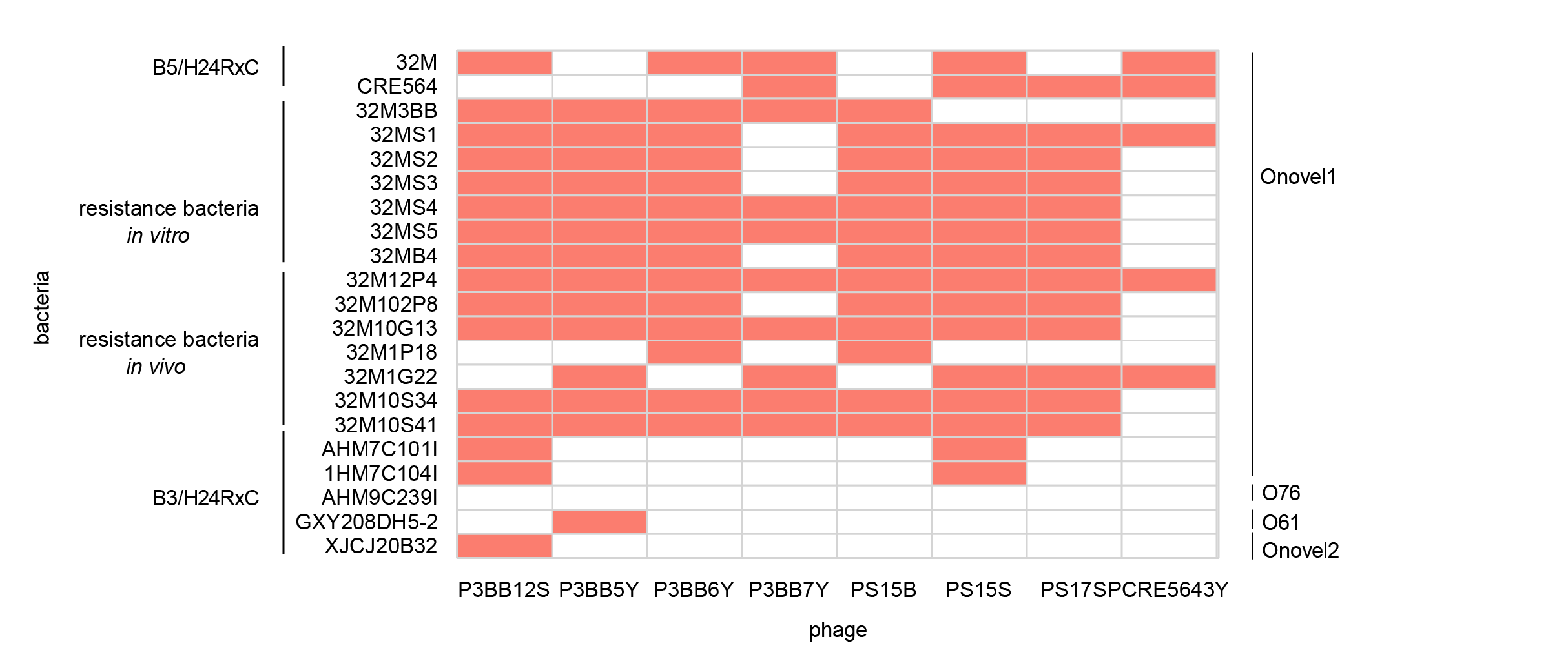

Supplement: S3 Fig — Red and white blocks represent with and without lysis effect, respectively. (TIF) [file ppat.1013807.s003.tif]

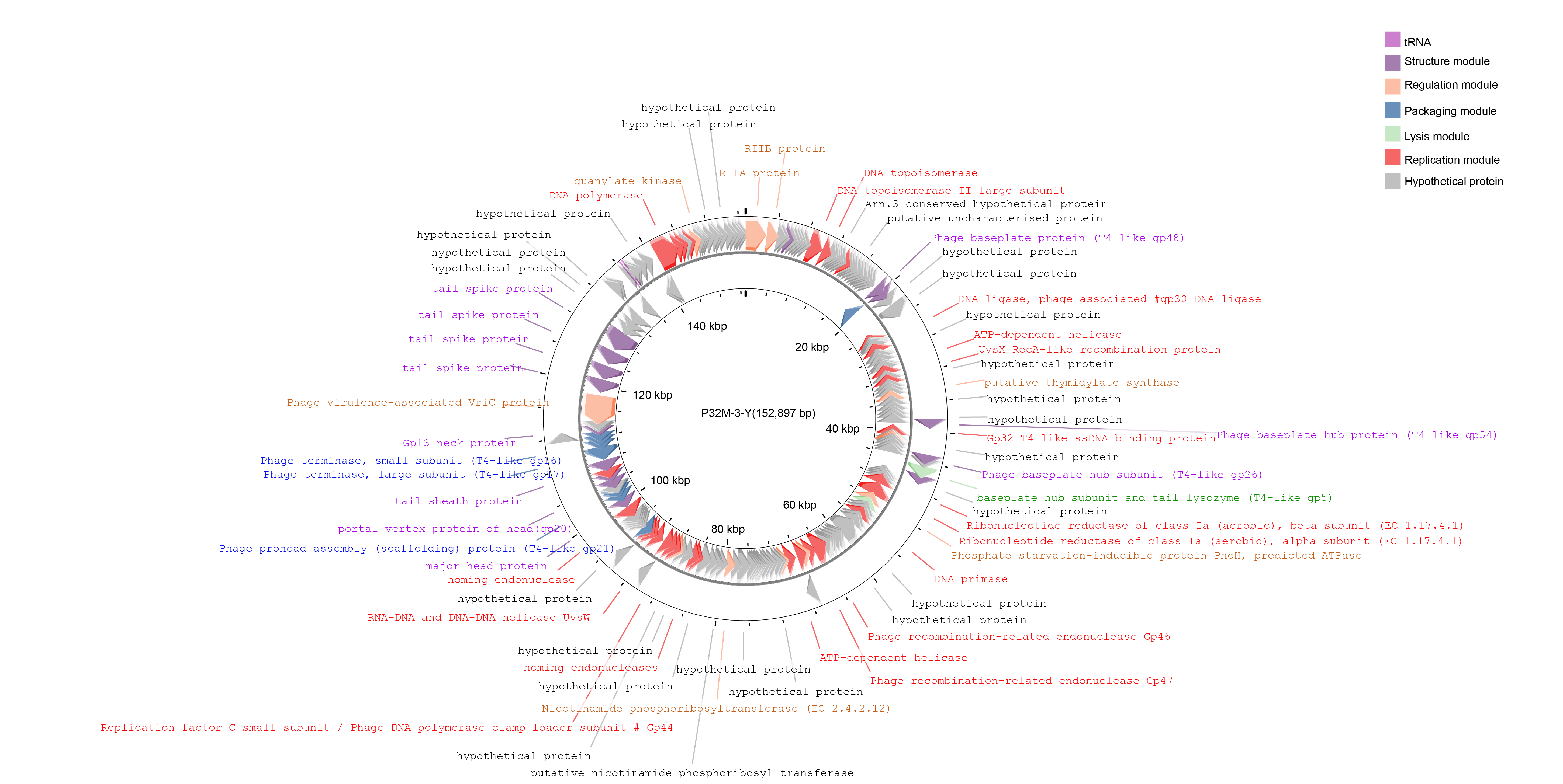

Supplement: S4 Fig — Different colored arrows represent predicted CDSs coding different functions: purple, structure module; yellow, regulation module; blue, packaging module; red, replication module; light green, lysis module; dark green, tRNA; grey, hypothetical protein. The genome map was generated using CGview Server. (TIF) [file ppat.1013807.s004.tif]

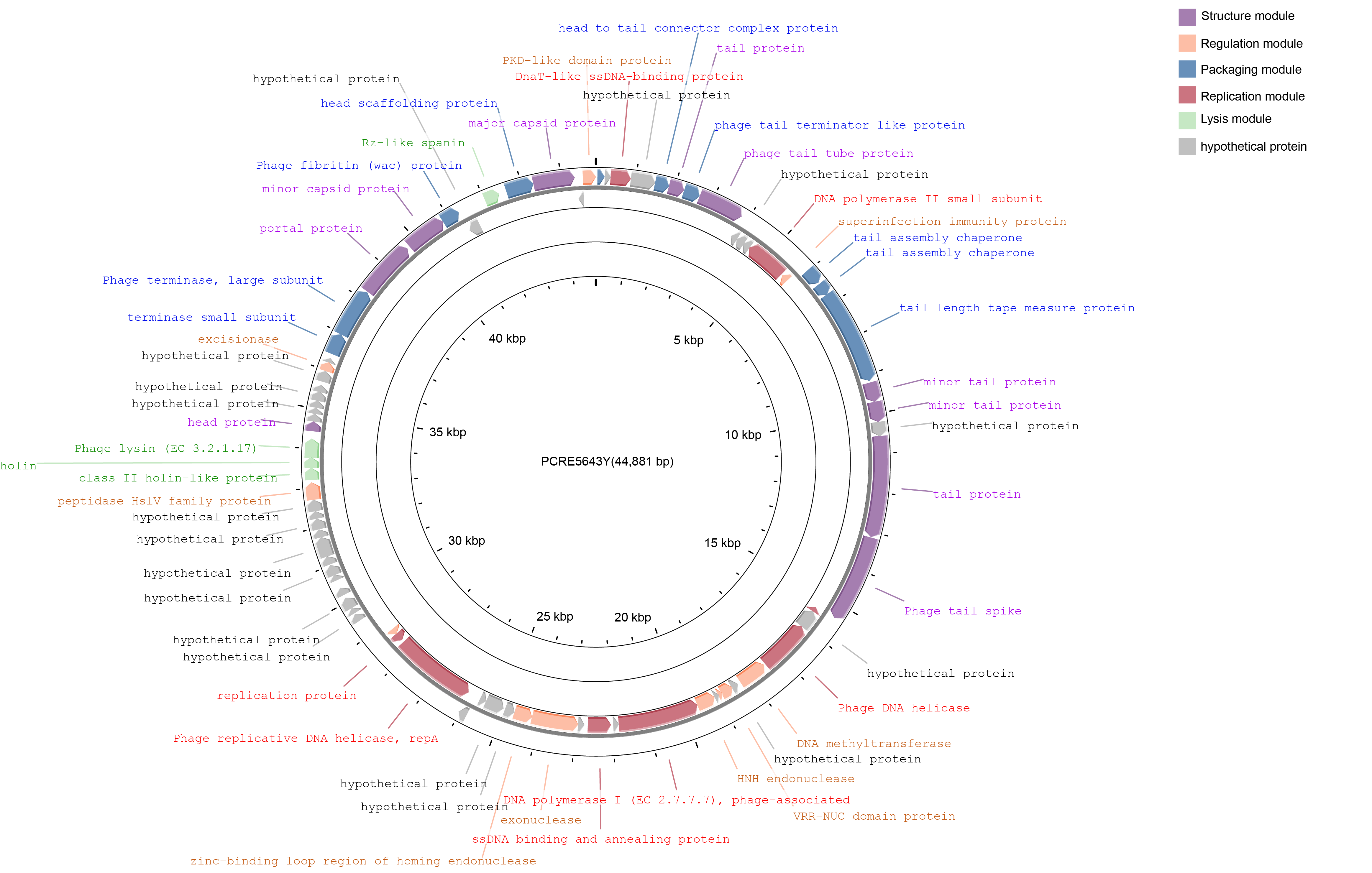

Supplement: S5 Fig — (TIF) [file ppat.1013807.s005.tif]

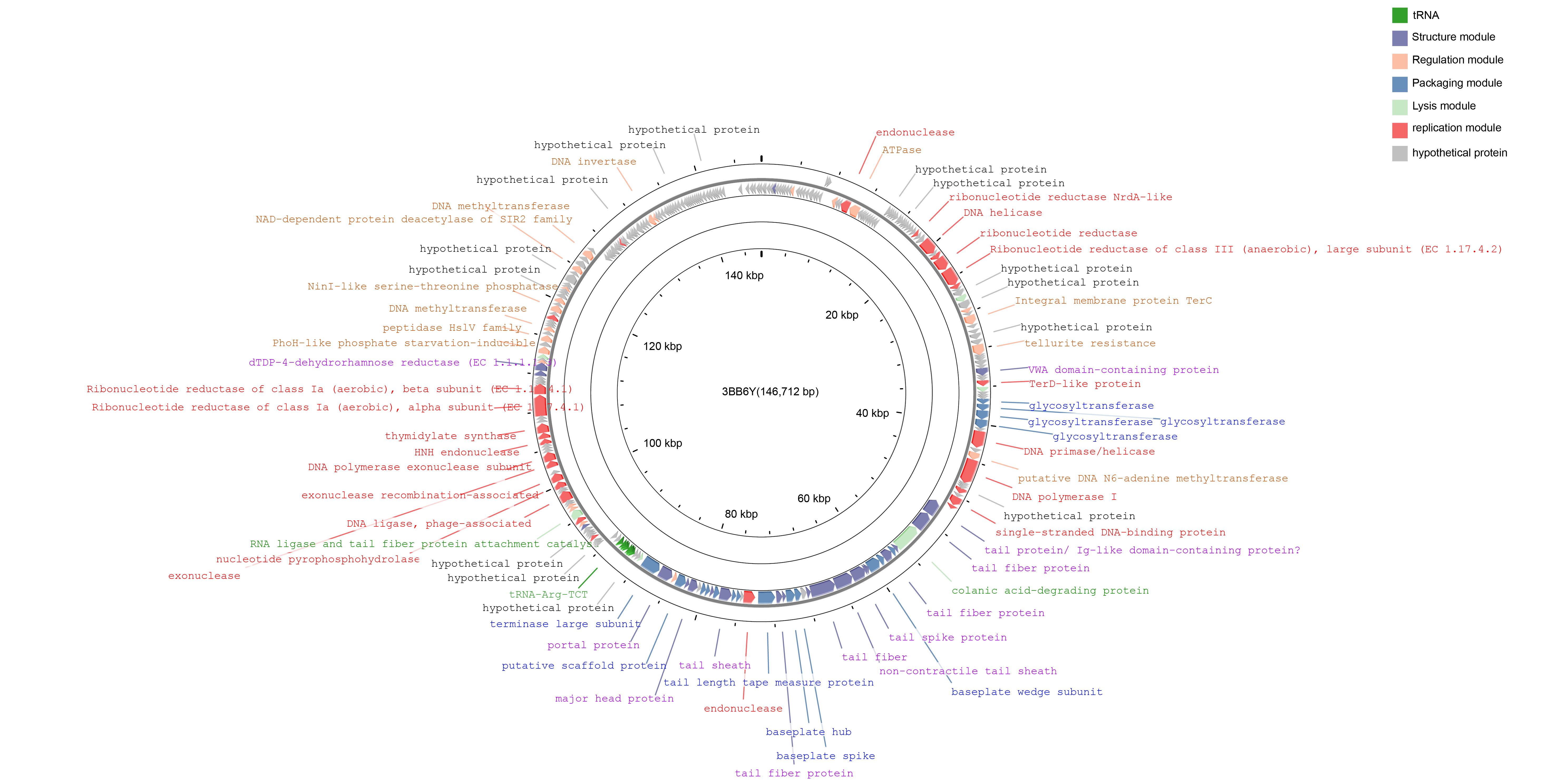

Supplement: S6 Fig — (TIF) [file ppat.1013807.s006.tif]

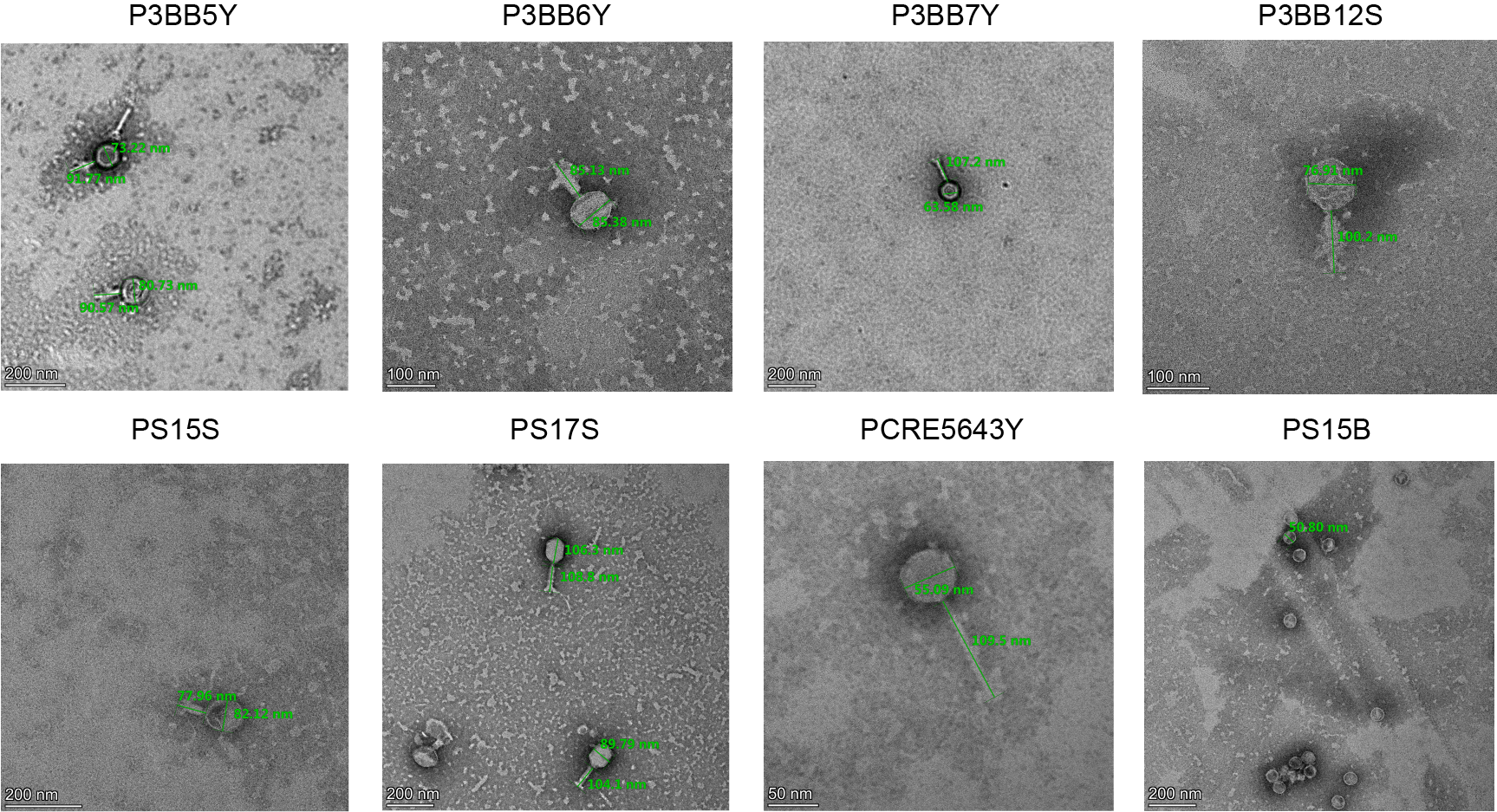

Supplement: S8 Fig — (TIF) [file ppat.1013807.s008.tif]
