# Supplementary material for: Colanic acid-mediated phage resistance enhances virulence in high-risk global clone Escherichia coli ST410
Source: PLoS Pathog. 2025 Dec 22;21(12):e1013807. doi: 10.1371/journal.ppat.1013807 (PMC12753057; doi:10.1371/journal.ppat.1013807)
Supplement: S1 Table — + , P-32M-3-Y could form a clear zone or plaque; -, P-32M-3-Y could not form a clear zone or plaque. (DOCX) [file ppat.1013807.s009.docx]

**S1 Table. The host range of phage P32M-3-Y against other ST *Escherichia coli*** +, P32M-3-Y could form a clear zone or plaque; -, P32M-3-Y could not form a clear zone or plaque.

| **ST** | **Strains** | **O-antigen** | **Lysis ability** | **ST** | **Strains** | **O-antigen** | **Lysis ability** |
| --- | --- | --- | --- | --- | --- | --- | --- |
| ST9124 | HZ9F01M | O3 | - | ST746 | AHM21C6767I | Untyped | - |
| ST57 | BY9F33M | O8 | - |  | AHM21C14634I | Untyped | - |
| ST206 | AHM9C61I | Untyped | - | ST9388 | AHM21C6603I | Untyped (O_9388) | + |
| ST1011 | AHM21C2872I | O11 | - |  | AHM21C5565I | Untyped (O_9388) | + |
| ST156 | AHM9C65WI | Untyped | - |  | AHM21C6661I | Untyped (O_9388) | + |
| ST195 | GD21SC1605T | O101 | - |  | AHM21C8639I | Untyped (O_9388) | + |
|  | GD21SC2376PT | O174 | - |  | AHM21C9158I | Untyped (O_9388) | + |
|  | GD21SC1970PT | O174 | - |  | AHM21C9158I | Untyped (O_9388) | + |
| ST167 | XJCJ20B23 | O101 | - |  | AHM21C14212I | Untyped (O_9388) | + |
|  | XJCJ21M70 | O101 | - |  | AHM21C14333I | Untyped (O_9388) | + |
