# Supplementary material for: Colanic acid-mediated phage resistance enhances virulence in high-risk global clone Escherichia coli ST410
Source: PLoS Pathog. 2025 Dec 22;21(12):e1013807. doi: 10.1371/journal.ppat.1013807 (PMC12753057; doi:10.1371/journal.ppat.1013807)
Supplement: S2 Table — (DOCX) [file ppat.1013807.s010.docx]

**S2 Table. Changes in antibiotic susceptibility of the phage-resistant strain 32M3BB.**

|  | CTX | IPM | CIP | AMK | FOS | TGC | DOX | FFC | CL |
| --- | --- | --- | --- | --- | --- | --- | --- | --- | --- |
| 32M | >128 | 4 | ＞64 | 2 | 4 | 0.125 | 16 | 4 | 0.125 |
| 32M3BB | >128 | 4 | ＞64 | 2 | 4 | 0.125 | 16 | 4 | 0.125 |
