# Supplementary material for: Colanic acid-mediated phage resistance enhances virulence in high-risk global clone Escherichia coli ST410
Source: PLoS Pathog. 2025 Dec 22;21(12):e1013807. doi: 10.1371/journal.ppat.1013807 (PMC12753057; doi:10.1371/journal.ppat.1013807)
Supplement: S3 Table — Cocktails were designed primarily based on differences in phage receptor usage to broaden host range coverage. The combinations shown represent a subset of possible designs. (DOCX) [file ppat.1013807.s011.docx]

**S3 Table Composition of phage cocktails used in Figures 4A and 4B.** Cocktails were designed primarily based on differences in phage receptor usage to broaden host range coverage. The combinations shown represent a subset of possible designs.

| Phage combinations | Phage names |
| --- | --- |
| Phage combination 1 | P-32M-3-Y, P-CRE5643Y, P-S17S |
| Phage combination 2 | P-32M-3-Y, P-CRE5643Y, P-3BB6Y, P-S17S, P-S15B |
| Phage combination 3 | P-32M-3-Y, P-CRE5643Y, P-3BB7Y, P-S17S, P-S15B |
| Phage combination 4 | P-32M-3-Y, P-CRE5643Y, P-3BB6Y, P-S17S |
| Phage combination5 | P-32M-3-Y, P-CRE5643Y, P-3BB6Y, P-S15B |
| Phage combination6 | P-32M-3-Y, P-CRE5643Y, P-3BB7Y, P-S17S |
