# Supplementary material for: Colanic acid-mediated phage resistance enhances virulence in high-risk global clone Escherichia coli ST410
Source: PLoS Pathog. 2025 Dec 22;21(12):e1013807. doi: 10.1371/journal.ppat.1013807 (PMC12753057; doi:10.1371/journal.ppat.1013807)
Supplement: S4 Table — (DOCX) [file ppat.1013807.s012.docx]

**S4 Table. The primer used in this study.**

| Primers | Sequences |
| --- | --- |
| yrfF-homo-up-F | catcgtgggatctatgctgtg |
| yrf-up-homo-R | ccgttCcgggtTatGgcAtgagtcagcatcatgacgttgttgc |
| yrfF-homo-down-F | tcaTgcCatAacccgGaacggcatctctaccgacgatc |
| yrfF-homo-down-R | gaccaggtcgataataccgagtg |
| yrfF-sgkp-F | acacacgctatcacgcgttagttttagagctagaaatagcaagtt |
| yrfF-sgkp-R | taacgcgtgatagcgtgtgtactagtattatacctaggactgag |
| RE-SGKP(RIP)-F | gtgatgacggtgaaaacctctg |
| RE-SGKP(RIP)-R | cagaggttttcaccgtcatcac |
| yrfF-F | ctgctcactacttgcgggatg |
| yrfF-R | accgttgagtgaaataaccag |
| cat-promoter-F | CCTTTTTGCGTTTCTACAAACTCTTtgatcggcacgtaagaggttc |
| pBAD-KO-AMP-R | AGAGTTTGTAGAAACGCAAAAAGG |
| pBAD-KO-AMP-F | ggcagttattggtgcccttaaacCTGTCAGACCAAGTTTACTCATATATAC |
| cat-R | gtttaagggcaccaataactgcc |
| yrfF-pBAD-F | CTAGCAGGAGGAATTCACCacacgcctgacagactaagtaagatg |
| pBAD24-R | tGGTGAATTCCTCCTGCTAG |
| yrfF-pBAD-R | TAGAGGATCCCCGGGTACCAAcagggtagcataacctgcc |
| pBAD24-F | TGGTACCCGGGGATCCTCTA |
| check-pBAD-F | GCATCAGACATTGCCGTCAC |
| check-pBAD-R | CTACTCAGGAGAGCGTTCACC |
| pBAD-Lon-F | CTAGCAGGAGGAATTCACCagtgtcatctgattacctggcgg |
| pBAD-Lon-R | TAGAGGATCCCCGGGTACCAAccgccatctaacttagcgag |
| lon-R | ccgccatctaacttagcgag |
| lon-F | gtcatctgattacctggcgg |
| check-SGspacer(RIF)-F | ctctcgtttggattgcaactgg |
| check-SGspacer(RIF)-R | ctttacactttatgcttccggc |
| q-cpsG-R | agtttgcccagcgtttttcc |
| q-cpsG-F | gcccaccactatttccgtga |
| q-rcsB-F | ctggcgataagtacggcgat |
| q-rcsB-R | gaatcgccgggttgttgttc |
| q-rcsA-F | gttgcgaatgtggatggcag |
| q-rcsA-R | atgcgatgaaacggtcttgg |
| q-wza-F | ctgactgaagctctgggcaa |
| q-wza-R | cgcctttcaacggacgaatg |
